# So Uncommon and so Singular, but Underexplored: An Updated Overview on Ethnobotanical Uses, Biological Properties and Phytoconstituents of Sardinian Endemic Plants

**DOI:** 10.3390/plants9080958

**Published:** 2020-07-29

**Authors:** Cinzia Sanna, Andrea Maxia, Giuseppe Fenu, Maria Cecilia Loi

**Affiliations:** 1 Department of Life and Environmental Sciences, University of Cagliari, Via Sant’Ignazio da Laconi 13, 09123 Cagliari, Italy; a.maxia@unica.it (A.M.); gfenu@unica.it (G.F.); loimc@unica.it (M.C.L.); 2 Co.S.Me.Se—Consorzio per lo Studio dei Metaboliti Secondari, Via Sant’Ignazio da Laconi 13, 09123 Cagliari, Italy

**Keywords:** Sardinia, endemic species, biodiversity, ethnomedicine, biological activities, secondary metabolites

## Abstract

The last decades have recorded an increase of plant-based drug discovery processes. Indeed, natural products possess a superior chemical diversity as compared to synthetic ones, leading to a renewal in searching for new therapeutic agents from the plant kingdom. In particular, since the structural variety of natural compounds reflects the biodiversity of their source organisms, regions of the world with high biodiversity and endemism deserve particular interest. In this context, Sardinia Island (Italy), with 290 endemic taxa (12% of the total flora), is expected to provide unique and structurally diverse phytochemicals for drug development. Several research groups built up a large program dedicated to the analysis of Sardinian endemic species, highlighting their peculiar features, both in respect of phytochemical and biological profiles. On this basis, the aim of this review is to provide an up-to-date and comprehensive overview on ethnobotanical uses, biological properties and phytoconstituents of Sardinian endemic plants in order to support their beneficial potential and to provide input for future investigations. We documented 152 articles published from 1965 to June 2020 in which a broad range of biological activities and the identification of previously undescribed compounds have been reported, supporting their great value as sources of therapeutic agents.

## 1. Introduction

Plants have been a valuable source of therapeutic agents for thousands of years, and their constituents have provided a substantial number of the natural product-derived drugs currently used in Western medicine [1,2]. Indeed, at least one quarter of the commonly used drugs are directly obtained or derived from plants [2]. This percentage considerably increases in particular areas, such as those of anticancer and antiviral drugs [2].

The plant-based drug discovery process reached its peak in the 1970–1980 decade, after which, in spite of its high value as a source of novel medicines, it has gradually decreased, because of the known perceived disadvantages and difficulties (e.g., availability of plant material, stricter regulations to safeguard and conserve plant diversity, seasonal and/or environmental variations of plant metabolites, high structural complexity of plant compounds, etc). Owing to these problems, and to the significant time-consuming and expensive compound isolation, many pharmaceutical companies have significantly scaled back or abandoned their natural product programs in favor of automated high throughput screenings (HTS) of synthetic compounds libraries [3]. Therefore, natural extracts were replaced with synthetic molecules. However, a small number of newly introduced drugs from an origin in HTS of combinatorial chemistry libraries have reached the market in the last years [3,4].

These disappointments, and the awaraness that natural products possess a superior chemical diversity as compared to synthetic ones, have led to a renaissance of the interest in searching for new plant-based drugs [5,6].

It is currently estimated that only approximately 6% of the plant kingdom has been screened for biologic activity, and only 15% for their phytochemical profiles [7], thus the vast majority of plants’ diversity is yet to be exploited. Since the chemical diversity of plant secondary metabolites can reflect the biodiversity of their source organisms, regions with high rates of endemisms could be particularly interesting in searching for new bioactive compounds, as is widely documented [8,9,10,11,12].

In this context, the Mediterranean Basin deserves particular interest, since it is widely recognized as one of the hot spots of terrestrial biodiversity [13,14,15]. This area, which corresponds to 2% of the world’s surface, is characterized by a high rate of endemism, with approximately 60% of all native taxa being Mediterranean endemics, and half of which correspond to endemic *sensu stricto* [13,16,17]. Nevertheless, this endemic plant richness is irregularly distributed [13,16,18]; in particular, endemic plant diversity occurs on the large Mediterranean islands and archipelagos that represent the principal centers of diversity having an endemism rate of more than 40%, particularly due to the restricted range of most of their flora [16,19].

Within Mediterranean islands, Sardinia (Italy), with its total area of approximately 24,000 km^2^, is the second largest island after Sicily [13,18]. Sardinian flora consists of 2441 taxa of native vascular plants [20]; out of them, 290 are endemic (183 taxa exclusive to Sardinia, 90 Sardo-Corsican and 17 also present in the Tuscan Archipelago) [18]. This endemic richness is attributable to the long geological history of the island, its prolonged geographical isolation and the high geomorphological diversity, especially on its mountain massifs, which have contributed to developing a wide range of habitats [13,18,21]. It is expected that this relative high rate of endemism provides a plethora of unique and structurally diverse phytochemicals for drug development. Consequently, in the last decades, several research groups have built up a large program dedicated to the analysis of Sardinian endemic species, highlighting their peculiar features, both in respect of the phytochemical profiles and biological potential.

On this basis, the aim of this review was to provide for the first time an up-to-date and comprehensive overview of ethnobotanical uses, chemical constituents and pharmacological properties of Sardinian endemic plants, in order to facilitate further investigations into the complete chemical profile of the secondary metabolites, as well as their pharmacological activities and molecular mechanisms.

## 2. Methodology

An in-depth research on the available literature was conducted on ethnobotanical uses, phytoconstituents and biological activities of Sardinian endemic plants. Information was mainly collected from international peer-reviewed papers found via online scientific databases, namely Medline (PubMed), Scopus, Web of Science, Researchgate and Google Scholar. As keywords, the Linnean binomial of each taxon were firstly used; in addition, a second query was also performed including their associated synonyms. Out of the articles found, only those regarding traditional uses, biological properties and phytochemical data were considered. The list of endemic species was obtained from Fenu et al. [18] for those plants growing in Sardinia, Corsica and Tuscan Archipelago. Moreover, other endemic plants, distributed in a wide area, were considered, according to the most relevant articles on Sardinian floras recently published [13,18,22,23].

In addition to the online-published papers, data on the usage of Sardinian endemic plants in traditional medicine were also sourced from paper articles and books. All articles have been published from 1965 to June 2020.

## 3. Results and Discussion

**Figure 1:**
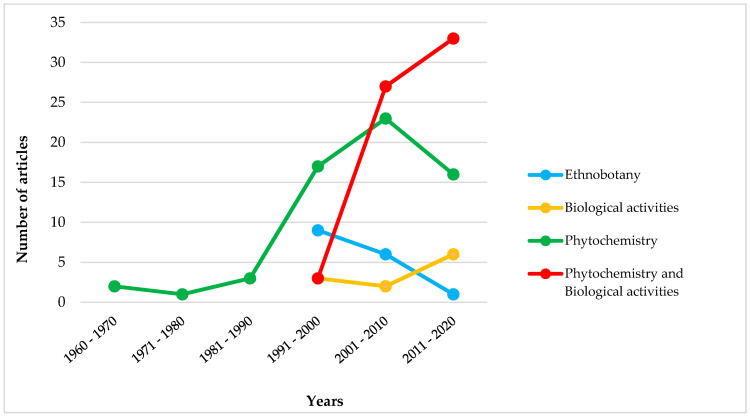
Number of articles on ethnobotanical uses, phytochemical features and/or pharmacological properties of Sardinian endemic species published from 1965 to 2020 (*n* = 152).

Our literature search led to one-hundred and fifty-two articles, covering fifty-one Sardinian endemic species (around 17% of total endemic flora) belonging to nineteen families and thirty-four genera. Out of articles on chemical analysis and biological evaluation, those which referred to samples collected in Corsica have also been found and then considered. As shown in Figure 1, the number of articles considerably increased in the last two decades, evidencing a great interest of the scientific community in searching for new compounds from unstudied or underexplored plants. In fact, biodiversity-rich regions, such as Sardinia, provide unique chemical scaffolds that could be used as templates for medicinal chemistry.

The first articles concerned the isolation and structural elucidation of secondary metabolites (Figure 1). Interestingly, they were published about thirty years before the first reports on uses of Sardinian endemic species for medicinal purposes. Indeed, ethnobotanical surveys in which Sardinian endemic plants were used by local inhabitants have been published since 1991. This suggests that ethnobotanical information did not play a key role in the plant selection for chemical studies. The number of articles regarding only chemical features has progressively decreased since 2001, and simultaneously, those in which the evaluation of biological activity is coupled with the identification of bioactive compounds increased. In fact, the interest for the therapeutic potential of Sardinian endemic plants started in the 1990s and has progressively intensified until today. Instead, the number of articles describing biological properties of crude extracts is considerably lower. Indeed, the complex mixtures produced from plants are perceived to be incompatible with high throughput screenings, because they often contain some components, such as tannins, which can produce false-positive results in both enzymatic and cellular assays [3].

The list of endemic plants that came to light in our review is shown in Table 1, together with their distribution range; specifically, twenty taxa are exclusive to the island, sixteen are present in Sardinia and Corsica, and nine are endemic of Sardinia, Corsica and Tuscan Archipelago. The remaining six taxa are recorded also in Sicily, Balearic Islands, other minor Mediterranean islands and islets, and along the Mediterranean coasts of the Italian Peninsula. Fabaceae, Asteraceae and Lamiaceae are the highest studied families.

### 3.1. Ethnobotanical Uses

Sixteen articles on traditional uses of Sardinian endemic species have been found. Ethnobotanical uses, along with information on the part(s) of plant used, type of preparation and places where they have been used, and their respective references, are reported in Table 2. 

Despite the rich and ancient Sardinian ethnobotanical tradition [24,25], only twenty-one endemic plants have been used in traditional medicine, probably because of their extreme rareness. In fact, largely used medicinal plants are usually common species, since they are abundant and available [26]. Dermatological problems, respiratory ailments and gastrointestinal conditions are the most frequent categories of use. Leaves, whole plant, underground organs (roots, rhizomes, tubers or bulbs), stems and flowers are the parts of plant more frequently used. With regard to the preparations, infusion and decoction stand out as the most used, followed by cataplasm or direct application. The latter are mostly prepared to treat skin diseases and as an anti-rheumatic. Peculiar references concern the use of *Scrophularia trifoliata* for the Basedow’s disease, an autoimmune disorder [27], and *Vinca difformis* subsp. *sardoa* as an antitubercular [28]. With the exceptions of *Cymbalaria muelleri* [29,30], *Glechoma sardoa* [29,30], *Ptilostemon casabonae* [30] and *Verbascum conocarpum* subsp. *conocarpum* [30], which have been used in just one territory, the others plants were largely employed by Sardinian inhabitants.

**Figure 2:**
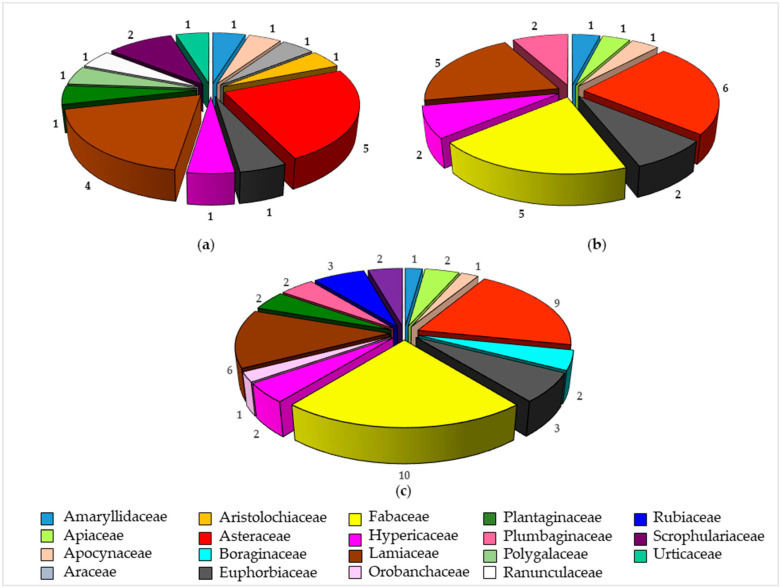
Overview of Sardinian endemic species, grouped by families, for which ethnobotanical (**a**), pharmacological (**b**) and phytochemical (**c**) data have been published (1965–2020).

It is noteworthy that, out of the twenty-one endemic species used in traditional medicine, sixteen have been subjected to phytochemical and/or pharmacological investigation, often exhibiting interesting properties, even though they are different from their uses in Sardinian ethnomedicine (see the paragraph *3.2 Pharmacological activities*). Indeed, to date, limited scientific studies have aimed at supporting their traditional uses. As shown in Figure 2, our literature search pointed out that the selection of endemic plants for extract screening has probably been achieved by different approaches, with no regard for the ethnobotanical information, according to what has been observed in Figure 1.

Indeed, even though the ethnomedicine has guided the field of bioscreening for a long time, often leading to the discovery of several plant-derived drugs [31], in the discovery of new drug leads from medicinal plants, chemotaxonomic and biodiversity-driven approaches can also be adopted [3]. The former allows for predicting that a plant is taxonomically related to others, which produce certain classes of natural products and may contain similar metabolites. Conversely, the biodiversity-driven approach, namely random screening, consists of selecting plants based on their availability [3].

Five endemic taxa, namely *Aristolochia thyrrena* (Aristolochiacee), *Arum pictum* subsp. *pictum* (Araceae), *Polygala sardoa* (Polygalaceae), *Staphisagria requienii* subsp. *picta* (Ranunculaceae) and *Urtica atrovirens* (Urticaceae) have not been subjected to further investigation, despite their acceptance and documented uses among the local inhabitants. Indeed, to the best of our knowledge, they lack chemical in-depth analysis and phytotherapeutic evidences. Conversely, species belonging to Fabaceae, Apiaceae, Boraginaceae, Orobanchaceae, Plumbaginaceae and Rubiaceae families have been selected for biological and phytochemical evaluation, even though they are not used in Sardinian ethnomedicine (Table 2).

**Table 2:** Ethnobotanical uses of Sardinian endemic species.

Taxon	Ethnobotanical Uses	Part(s) of the Plant Used	Preparation	Territories	References
*Aristolochia thyrrena*	Emmenagogue, vulnerary	WP	Infusion	Barbagia Seui, Seulo, Sadali	[30,32,33]
*Arum pictum* subsp. *pictum*	Vulnerary, burns and scalds	L, T	Cataplasm	Villagrande Strisaili, Barbagia Seui, Urzulei, Barbagia Seulo	[30,32,33,34,35]
	Diuretic, nervous sedative	L	Decoction	Urzulei	[30,34]
	Haemostatic	L	Direct application	Tempio Pausania	[30,36]
	Anti-rheumatic	F	Cataplasm	Urzulei, Barbagia Seui, Barbagia Seulo	[30,32,34]
	Skin lightening	T	Latex	Villamassargia	[30]
*Cymbalaria muelleri*	Burns, chilblains, skin inflammation, hemorrhoids	AP	Cataplasm	Laconi	[29,30]
*Euphorbia pithyusa* subsp. *cupanii*	Removal of warts	LX	Direct application	Barbagia Seui, Sadali, Seulo	[30,32,33]
	Antiasthmatic	FP	Decoction	Barbagia Seui, Sadali, Seulo	[30,32]
	Analgesic	LX	Direct application	Bolotana	[30]
*Glechoma sardoa*	Respiratory diseases, chronic catarrh, bronchitis, antiasthmatic, wound healing	F and S	Infusion with milk and honey	Laconi	[29,30]
	Vulnerary, resolvent in scalds, antinevralgic, antirheumatic	F and S	Infusion, infusion with milk and honey (direct application)	Laconi	[29,30]
*Helichrysum italicum* subsp. *tyrrhenicum*	Anti-allergic	WP	Infusion	Urzulei	[34]
	Skin diseases (alopecia), diaphoretic, burns, vulnerary	WP, FH, S	Decoction, infusion, cataplasm	Fluminimaggiore, Gesturi, Tempio Pausania	[27,36,37]
	Bronchitis, laryngitis, tracheitis, cough sedative, expectorant	L, FH	Infusion	Barbagia Seui, Laconi, Escolca, Tempio Pausania	[29,32,36,38]
	Antineuralgic, antirheumatic	FH	Infusion	Barbagia Seui, Laconi, Tempio Pausania	[29,32,36]
*Hypericum hircinum* subsp. *hircinum*	Burns and wound healing	F	Macerate in olive oil	Laconi	[29,30]
	Antirheumatic, sciatica, dislocation	F	Macerate in olive oil and wine	Laconi	[29,30]
	Balsamic, antiasthmatic	WP	Infusion	Laconi	[29,30]
*Pancratium illyricum*	Depurative, diuretic, emetic, corn removal, antiseptic	B	Powder, cataplasm	Tonara, Iglesiente	[30]
*Polygala sardoa*	Fluidifyng	R	Decoction	Laconi	[29,30]
*Ptilostemon casabonae*	Antispasmodic	AP	Ingestion	Urzulei	[30]
*Salvia desoleana*	Antipyretic, anti-inflammatory	S and L	Decoction	Loceri	[30]
	Vulnerary	L	Cataplasm	Loceri	[30]
	External anti-inflammatory	L	Heated in oil	Villanovaforru	[30]
*Santolina corsica*	Anthelmintic, tonic, emmenagogue, insect repellent	AP	Not available information	Lodè, Lula	[30]
*Santolina insularis*	Insect repellent (pediculus)	WP, S, L	Fumigation	Arzana, Villagrande Strisaili	[35,39]
	Anthelmintic	FH, L	Infusion	Marganai, Laconi	[29,40]
	Febrifuge, sedative, antitussive	L	Decoction	Villagrande Strisaili	[35]
*Scrophularia trifoliata*	Skin diseases, vulnerary, anti-edema, antirheumatic	L, FH, RH	Infusion, cataplasm of fresh leaves with olive oil, cream	Ussassai, Urzulei, Villagrande Strisaili, Escolca	[28,33,34,35,38]
	Diuretic	L	Decoction	Escolca	[38]
	Emollient	R	Direct application	Barbagia Seui, Sadali, Seulo	[30,32,33]
	Anthelmintic	R	Powder with honey	Barbagia Seui, Sadali, Seulo	[30,32]
	Purgative, emetic, Basedow’s disease and related heart disorders	L	Infusion	Gesturi	[27]
	Antirheumatic, vurnerary	L	Cataplasm	Urzulei	[30]
	Antipyretic, anti-inflammatory, tonsillitis, sore throat	L	Cataplasm	Aggius	[30]
*Stachys glutinosa*	Hepatoprotective, cholagogue, diuretic	L	Decoction	Gesturi	[27]
	Common cold	L	Decoction	Villagrande Strisaili	[35]
	Antiseptic, antispasmodic	WP	Infusion	Arzana	[30,33,39]
	Sedative	WP	Infusion	Bolotana	[30]
*Staphisagria requienii* subsp. *picta*	Antiparasitic (lice, nits, mites), vulnerary	L, SE	Ointment, powder	Barbagia Seui, Bolotana	[30,32]
*Tanacetum audibertii*	Digestive, anthelmintic, anti-arthritic, emmenagogue	AP	Decoction	Not available information	[30]
*Thymus herba-barona* subsp. *herba barona*	Anthelmintic, digestive, to treat gastralgia	WP, S, L, R	Decoction, infusion	Arzana, Urzulei, Laconi	[29,34,39]
	Antirheumatic	WP	Cataplasm	Urzulei	[34]
	Cough sedative, whooping cough, expectorant	L, F	Infusion with milk, decoction	Arzana, Ussassai, Laconi	[28,29,39]
	Antipyretic	WP	Decoction with malva and lemon, soaked in “grappa”	Arzana, Urzulei	[34,39]
	Sore throat, common cold, cough, bronchitis, asthma, tonic, antianaemic, intestinal antispasmodic, diuretic	L, S	Decoction, inhalation of infusion with malva, rosemary and sage	Arzana, Villagrande Strisaili, Laconi, Urzulei, Perdasdefogu	[29,34,35,39,41]
	Cold	R and F	Inhalation of decoction	Arzana	[39]
	Antiseptic, tonic, disinfectant, mouthwash	L, L and R	Infusion, decoction	Perdasdefogu, Arzana	[33,39,41]
	Urticaria, foot perspiration	WP	Powder	Perdasdefogu	[33,41]
	Lenitive	L	Cataplasm	Ussassai	[28]
	Toothache	R	Chewing	Arzana	[39]
*Urtica atrovirens*	Baldness, rashes, vulnerary, hemostatic, antirheumatic, emmenagogue, gastralgia	L, WP	Infusion	Lotzorai, Marganai, Domusnovas	[30,38,40]
	Diuretic	WP, L	Infusion, syrup	Barbagia Seui, Marganai, Seulo, Sadali	[30,32,40]
*Verbascum conocarpum* subsp. *conocarpum*	Anti-inflammatory, anti-catarrhal	L	Decoction, fumigation	La Maddalena Archipelago	[30]
*Vinca difformis* subsp. *sardoa*	Anti-rheumatic	L	Application of heated leaves	Ussassai	[42]
	Sedates nausea	L	Infusion	Escolca	[38]
	Anti-emetic, eupeptic, anti-inflammatory, anti-hemorragic, galactofuge, astringent, hypotensive, hypoglycaemic	L	Decoction, infusion	Laconi, Perdasdefogu, Escolca, Monteleone	[29,38,41,42]
	Antitubercular	L	Infusion, maceration	Monteleone	[42]
	Bronchitis	L	Poultice	Lodine	[43]

In the column “Territories”, localities, municipalities and/or regions where each plant has been used were reported. The part(s) of the plants used have been abbreviated as follows: L (leaves); F (fruits); B (bulbs); WP (whole plant); S (stem); R (roots); F (flowers); FH (flower head); LX (latex); RH (rhizomes); T (tuber); AP (aerial parts); FP (flowering plant); SE (seeds).

### 3.2. Pharmacological Activities

**Figure 3:**
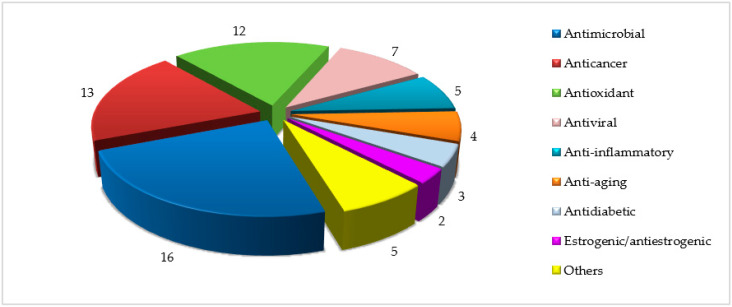
Number of Sardinian endemic species grouped by their documented biological activities.

Overall, seventy-four scientific articles, in which essential oils, crude extracts and/or pure compounds isolated from twenty-five Sardinian endemic species have been evaluated for their therapeutic potential, were found in the literature (Supplementary Table S1). Several categories of biological activities have been identified and the most represented were those of antimicrobials, anticancers, antioxidants and antivirals (Figure 3). All these categories are discussed, item by item, below.

#### 3.2.1. Antimicrobial Activity

Since their discovery, antibacterial drugs have become an essential part of the present healthcare scenery, even though their incorrect use or abuse cause the growth and spread of microorganisms resistant to their action, with consequent loss of efficacy of the therapies. Indeed, antimicrobial resistance (AMR) is nowadays recognized as a global public health problem [44]. Consequently, in this scenario, there is an urgent need of new effective antifungals and antibacterials without undesirable side effects, and plants could give a very important contribution [45].

Our survey led to sixteen Sardinian endemic plants with antimicrobial properties.

Essential oils of *Artemisia caerulescens* subsp. *densiflora* and *Mentha requienii* subsp. *requienii* showed antifungal properties against environmental isolates of yeasts and molds strains (*Rhodotorula* spp., *Candida* spp., *Aspergillus* spp., *Alternaria* spp. and *Fusarium* spp.), suggesting their possible use as alternatives to the common disinfectants and also as post-harvest control of cereals [46,47]. N-hexan extract obtained from *Astragalus verrucosus* exhibited antibacterial activity against Gram+ bacteria (*Staphylococcus aureus*), while polar extracts, especially the butanol one, showed antifungal activity against *Aspergillus niger* and *Botrytis cinerea* [48]. The activity of the polar extract could be attributable to the flavonoid compounds [49]. In addition, the new saponin isolated from this plant, namely astraverrucin II, showed a significant antifungal effect, maybe due to the presence of an acetyl group. Indeed, the other astraverrucins lacking of this group were not active [48].

Ethyl acetate extract of whole plant and the acetonitrile extract of latex of *Euphorbia semiperfoliata* were able to inhibit the multidrug transporter CaMdr1p of *Candida albicans*. In particular, the antibacterial activity of a diterpenoid ester seems to be related to the presence of an isobutyrate group at C-8, instead of an acetoxy or hydroxyl group [50]. The essential oil of *Glechoma sardoa*, studied by the agar dilution method, was effective against Gram+ bacteria, such as *S. aureus* and *S. epidermidis*, but no activity was observed on Gram– bacterial strains [51]. *Helichrysum italicum* subsp. *thyrrenicum* showed a strong antimicrobial activity: both the essential oil and methanol extract exhibited antifungal properties against *Candida* ssp. and *Pythyum ultimum*, the latter is a phytopathogen [52,53,54]. Interestingly, the anticandidal activity of the oil is enhanced by sub-inhibitory concentrations of chitosan, suggesting its use in innovative formulations, as a therapeutic alternative in the treatment of *Candida* opportunistic infections [53]. It is worth noting that it is well-known that the antimicrobial activity of *Helichrysum* ssp. is generally attributed to nerol esters [55,56]; however, in Sardinian samples, the antibacterial activity was observed in the chemotype rich in rosifoliol but missing nerol and its esters [52]. The methanol extract is also active against the dermatophyte *Trichophyton mentagrophytes* var. *mentagrophytes* and against Gram+ bacteria, such as *Micrococcus luteus* [54]. Interestingly, the heterodimeric phloroglucinol α-pyrone arzanol exhibited a significant antibacterial activity against multidrug-resistant *S. aureus* isolates [57]. In particular, it was active on bacterial strains, which overexpress the NorA pump [57], a transporter able to efflux a wide range of antibiotics [58]. The oil macerate of aerial parts was found to be active against *Candida* spp. clinical isolates [59]. All these results validated the topical use of *Helichrysum* extracts to prevent wound infections and treat skin diseases in traditional medicine [37].

Recently, a remarkable activity of *Limonium morisianum* towards multidrug-resistant *S. epidermidis* clinical isolates has been described as well as the correlation among total phenolic content and increasing antibacterial activity [60].

The antibacterial activity of ungeremine, an alkaloid extracted from bulbs of *Pancratium illyricum* able to impair both human and bacterial (*Escherichia coli*) topoisomerases, was recently described [61]. These enzymes are two new interesting targets in antimicrobial chemotherapy. Moreover, the alkaloid extract of bulbs, and especially lycorine, was identified as the major active component with a potent inhibitory effect on *C. albicans* clinical isolates [62]. On the contrary, lycorine did not exert antibacterial activity, probably because of the ability of some bacteria, such as *S. aureus*, to transform it in its inactive metabolite 2-O-demethylungiminorine, instead of the active one, ungeremine [63].

Both essential oil and supercritical fluid extract from *Plagius flosculosus* exhibited antifungal properties against *C. albicans* and *Mycobacterium smegmatis* [64]: since the antimicrobial concentrations were generally higher than cytotoxic doses, they can be considered interesting as antimicrobial drugs on superficial microbial infections.

The essential oil of *Salvia desoleana* possesses antifungal properties against several mycromycetes. The activity probably derived from the synergistic action between different oil components, since the main components, such as linalyl acetate, 1,8-cineole and linalool, showed a slight antifungal activity [65]. Furthermore, even if it exerted a weak inhibition activity against *S. aureus*, *S. epidermidis*, *E. coli* and *C. albicans*, this inhibition increased progressively with contact time [66].

Both *Santolina corsica* and *Santolina insularis* exhibited antimicrobial properties. All studies regarding the first species are referred to as Corsican samples and they documented the antibacterial activity of the essential oil, probably due to the content of lyratol and 1,8-cineol in the oxygenated fraction [67,68]. Indeed, these compounds were capable of affecting the integrity of *S. aureus* plasma membrane and cell wall [69]. The hydrodistillated and supercritical fluid extract of *S. insularis* exerted antibacterial and antifungal activities against dermatophytes and yeast, especially *Cryptococcus neoformans* [70,71]. Furthermore, the essential oil decreased the germ tube formation in *C. albicans*, making it a promising anti-*Candida* agent [71]. The essential oil was active against some Gram+ bacteria and its effect, probably attributable to artemisia ketone and β-phellandrene [72], is enhanced by sub-inhibitory concentrations of chitosan [73]. Castangia and coauthors [74] reported that the essential oil of *S. insularis*, incorporated in liposomes, improves its delivery to the skin.

The essential oil of *Stachys glutinosa* exhibited good bacteriostatic activities against *Vibrio cholera, Candida glabrata* and *Rodotorula rubra* clinical strains, and bactericidal activities against the last two yeasts [75]. The hydrodistillated of *Tanacetum audibertii* exerted antifungal activity against yeasts and dermatophytes strains, especially on *C. neoformans* [76]. It showed a lower effectiveness against *Candida* ssp. in the macrodilution broth method, even though an evident inhibitory effect on germ tube formation in *C. albicans* was observed [76].

The essential oil of *Thymus herba-barona* subsp. *herba-barona* was active against *Candida* ssp., *C. neoformans*, dermatophytes and *Aspergillus* ssp. strains, even though it was cytotoxic on macrophages [77]. Due to its ability to inhibit *Aspergillus* growth, it could be used as a preservative in storage products. The oil was also able to inhibit the growth of a panel of standard reference and multiple strains of food-derived spoilage and pathogenic bacteria, due to its content in carvacrol and thymol [67,78,79].

#### 3.2.2. Antiviral Activity

The area of infectious diseases has benefited from abundant chemical compounds isolated from plants [80]. Indeed, despite the fact that the number of approved antivirals has considerably increased, these drugs are not always efficacious, and drug-resistant virus strains are rapidly emerging. Our search led to the following seven Sardinian endemic plants endowed with antiviral activities: *Euphorbia semiperfoliata*, *Helichrysum italicum* subsp. *thyrrenicum*, *Hypericum hircinum* subsp. *hircinum*, *Hypericum scruglii, Limonium morisianum*, *Salvia desoleana* and *Santolina insularis*. The first five exhibited a significant anti-HIV-1 (Human immunodeficiency virus type 1) activity, acting by different mechanisms of action. Some new jatrophane diterpenes and 4-deoxyphorbol esters isolated from *E. semiperfoliata* interfered with viral entry by inducing downregulation of HIV receptors in a virus-cell-based assay [81,82]. The same compounds were also able to inhibit the replication of Chikungunya virus (CHIK), probably involving, in both cases, a protein-kinase C-dependent mechanism, despite their different replication strategies [81,82]. The anti-HIV-1 activity of *H. italicum* subsp. *thyrrenicum* could be attributable to arzanol, which exerted its antiviral activity by inhibiting the transcription nuclear factor-kB (NF-kB), a validated target for inflammation, also involved in several other pathologies, including AIDS (Acquired Immune Deficiency Syndrome) [83]. Some active constituents isolated from *H. hircinum* subsp. *hircinum*, such as betulinic acid and two flavanone derivatives, showed a strong inhibition of both DNA polymerase and Ribonuclease H activities associated to HIV-1 reverse transcriptase (RT) enzyme [84]. In particular, betulinic acid, a triterpene compound already known for its antiviral activity [85], was also active on HIV-1 mutant RTs resistant to efavirenz [84]. Recently, the anti-HIV-1 activity of some phloroglucinol derivatives isolated from aerial parts of *H. scruglii* has been described [86]. In this case, a previously undescribed acylphloroglucinol was able to inhibit the virus replication. Results of time of addition experiments were compatible with an action on RT, while the anti-integrase (IN) activity exhibited in enzymatic assays was not significantly involved in the inhibition of viral replication. The presence of phlorogucinols in *H. scruglii* has confirmed their importance as chemotaxonomic markers of the *Hypericum* genus [87].

Among the secondary metabolites isolated from *L. morisianum*, (-)-epigallocatechin 3-O-gallate (EGCg) and myricetin-3-O-(6″-O-galloyl)-β-d-galactopyranoside potently inhibited both Ribonuclease H (RNase H) and Integrase (IN) functions in enzymatic assays [88]. The presence of a galloyl moiety in both compounds could explain their anti-HIV-1 activity, according to previous reports [89]. This species was also identified as a potent inhibitor of Ebola virus, since the flavonoid myricetin isolated from its aerial parts was capable of inhibiting the interaction between the viral protein VP35, which plays a fundamental role in the suppression of the host antiviral innate immune response, and the viral double-stranded RNA [90].

The essential oils of *S. desoleana* and *S. insularis* were found to be able to inhibit the herpes simplex virus-2 (HSV-2). In the first case, it inhibited both acyclovir-sensitive and -resistant HSV-2 strains by acting after virus attachment and entry [91]. Differently, the antiviral activity of *S. insularis* was principally due to a direct inactivation of virions, before its adsorption to host cells [92]. The essential oil of *S. insularis* also inhibited, through the same mechanism of action, the herpes simplex virus-1 (HSV-1). Furthermore, its incorporation in liposomes improved the oil stability and was demonstrated to be effective in inactivating HSV-1 [93].

#### 3.2.3. Anticancer Activity

Cancer represents a major undertaking that we are facing on a global health level. In 2018, 18.1 million people all over the world have had cancer, with a mortality rate of 53%. Unfortunately, these numbers are expected to significantly increase in the next twenty years [94]. Although prevention is indubitably the best approach, there are different lines of attack for cancer treatment, from surgery, radiotherapy, to chemotherapy and immunotherapy; unfortunately, they are often accompanied by side effects that have a negative impact on patients, undermining progress towards healing [95]. At present, more than half of the approved antitumor drugs have been isolated from plants and many evidences have demonstrated the potential of plant-derived compounds in both cancer prevention and treatment [96].

Among Sardinian endemic plants, the essential oils of *A. caerulescens* subsp. *densiflora* and *H. italicum* subsp. *tyrrhenicum* were evaluated on human malignant melanoma (A375), breast adenocarcinoma (MDA-MB-231) and colon carcinoma (HCT116) cell lines, exhibiting a concentration-dependent inhibitory effect on all human tumor cells, mainly on A375 [97,98]. Among the volatile compounds isolated from *A. caerulescens* subsp. *densiflora*, terpinen-4-ol and (E)-nerolidol were previously reported for their cytotoxic activity [99,100], but their concentrations in the oil did not explain the high cytotoxicity observed. Cytotoxic activity of *H. italicum* subsp. *thyrrenicum* is probably attributable to a synergistic action of various components of the essential oil, since to the best of our knowledge, there is no literature data on the cytotoxicity of the main compounds identified. Arzanol and methylarzanol, the latter obtained by methylation of the pyrone moiety, significantly reduced cancer Caco-2 cells’ viability at lower dosages [101]. It is noteworthy that arzanol selectively reduce viability of other several cancer cell lines, suggesting an action as a selective modulator of cell processes typical of cancer cells [101].

Petroleum ether and ethyl acetate extracts from leaves of *Bituminaria morisiana* showed cytotoxic activity against KB-(HeLa) (KERATIN-forming tumor cell line HeLa) and immunocompetent Jurkat T-cells due to the pterocarpans, especially erybraedin C [102]. The comparison between the activities of all isolated pterocarpans suggested that the prenyl chain at C-8, as well as free hydroxyl groups at positions 3 and 9 in the pterocarpan nucleus, enhanced cytotoxicity [102,103]. Furthermore, the activity of erybraedin C was not influenced by the overexpression of the mitochondria protecting protein Bcl-2, suggesting that it acts against Jurkat T cells via a mechanism other than the mitochondrial apoptotic pathway. Besides, the cytotoxic activity of this compound seems to be related to an induction of necrosis and not apoptosis [102]. A new prenylated pterocarpan, namely morisianine, obtained from *B. morisiana* seeds did not show any activity against Jurkat T, HL-60 (human leukemia), CaCo-2 (human epithelial colorectal adenocarcinoma) and MCF-7 (breast cancer) cell lines, up to the highest tested concentration [103].

Three macrocyclic jatrophane polyesters isolated from *E. semiperfoliata* induced tubulin polymerization into microtubules in vitro and inhibited the growth of some human cancer cell lines influencing p53 expression and Raf-1/Bcl-2 activation. However, differently from paclitaxel, they did not arrest cell cycle in the G2/M (Mitotic gap 2) phase [104].

Some daucane esters from *Ferula arrigonii* showed antiproliferative activity on several human colon cancer cell lines, in a dose-dependent manner. In particular, a new compound, 2α-OH-ferutidin, and the known ferutidin were the most active ones, followed by lapiferin and jaeskeanadiol [105]. All compounds have been previously reported in this species [106]. 2α-OH-ferutidin was found to be able to induce G0/G1 block into S phase of the cell cycle. Interestingly, this arrest was not followed by cell apoptosis, since the cell growth-inhibitory effect was reversible upon removal of the compound [105]. Moreover, the growth inhibitory potential of daucane esters was found to be positively correlated to their affinities for type II estrogen-binding sites (EBS) [105], which are present in many cancers [107]. In fact, 2α-OH-ferutidin and ferutidin, differently from lapiferin and jaeskeanadiol, were able to interact with type II EBS in WiDr (human colon adenocarcinoma) cells [105].

The alkaloid ungeremine, isolated from the bulbs of *P. illyricum*, was found to be able to impair both human and bacterial topoisomerases [61], two enzymes present in all living organisms, that control the topology of DNA in all cells and play a role in the cell replication [108]. Therefore, topoisomerases have become important targets for anticancer drugs. Interestingly, ungeremine was capable of incrementing the DNA cleavage stimulated by bacterial topoisomerase IA [61].

Several diacetylenic spiroketal enol ethers identified in the leaves of *P. flosculosus* showed significant cytotoxic activity against Jurkat T and HL-60 leukemia cell lines [109]. The activity was positively correlated with the presence of double bonds in the tetrahydrofuran ring, since compounds containing only one were less active. The most active compounds were also able to induce apoptosis in HL-60 cells in a concentration-dependent manner [109].

N-hexane and methanol extracts of *Santolina corsica* exhibited anti-proliferative activity on uterine cervical (HeLa), alveolar (A549), prostate (PC3), luminal and basal breast (MCF7 and MDA-MB-231) cancer cell lines [110]. This activity could be attibutable to the presence of some compounds, such as α-pinene [111] and limonene [112], as well as flavonoids [113], quercetin [114], known for their anti-proliferative effects. It is worth noting that anti-proliferative activities were specific for cancer cells, since neither of the two extracts induced cytotoxicity in the MCF10A non-tumorigenic breast epithelial cell line [110]. Furthermore, both extracts were able to reduce the metastatic capability of MDA-MB-231 breast cancer cells by reducing cells’ motility, migration and invasion [110]. Both n-hexane and methanol extracts could induce apoptosis, since after treatment with them, MDA-MB-231 cells changed their morphology, when compared to control cells [115].

Two germacrane sesquiterpenes isolated from *S. insularis* aerial parts showed a potent and selective cytotoxic activity against the human colon carcinoma cell line (Caco-2). In fact, both compounds resulted inactive on peritoneal macrophages [116]. It is worth noting that even though the terpenoid core on cytotoxic sesquiterpene exomethylene γ-lactones has been recognized to exert a significant role [117], the activity of compounds isolated from *S. insularis* attested that the sesquiterpene moiety is sufficient to explain the cytotoxic activity.

Ethanol extract of *S. glutinosa* exhibited a dose-dependent antiproliferative activity on human hepatocarcinoma (HepG2) and breast adenocarcinoma (MCF7) cell lines, while it was less effective against non-tumor muscle cells (C2C12, mouse myoblast), showing a selective activity [118].

The hydroalcoholic extract of *T. audibertii* exerted an interesting antitumor potential against human U2OS osteosarcoma cells [119], by blocking cell population in G2/M phase and affecting the tumor suppressor p53 levels, such as several anticancer agents [120]. Moreover, *T. audibertii* caused a significant activation of effector caspases [119], suggesting apoptotic cell death [121]. A strong cytotoxicity, also on venous endothelial cells (HUVEC), was recently reported [119], highlighting a potential use in the prevention of angiogenesis, an attractive target for cancer chemotherapy [122].

The essential oil of *T. herba-barona* subsp. *herba-barona* exhibited a high toxicity on a mouse macrophage cell line, even at lower concentrations, limiting its potential use for pharmaceutical and cosmetic purposes [77].

In searching for natural compounds with anticancer properties, anti-genotoxic and anti-mutagenic agents must also be considered, along with cytotoxic, anti-proliferative and pro-apoptotic agents. In fact, some anti-genotoxic compounds decrease the side effects associated with the most commonly used anticancer agents [123,124]. The flavonoid licoflavone C, isolated from the aerial parts of *Genista ephedroides*, was able to attenuate the effects of two mutagenic anticancer drugs, namely mitomycin C and daunorubicin, proving to be protective toward the chromosome damage in cultured human peripheral lymphocytes [125]. Licoflavone C, differently from the correspondent non-prenylated flavone apigenin, does not induce genotoxic activity [126], suggesting the key role of the prenyl group at C-8 in the A ring.

#### 3.2.4. Antioxidant Activity

Antioxidants, also called free radical scavengers, are all substances that inhibit oxidation and reduce the occurrence of cancer, diabetes, inflammation, cardiovascular and neurodegenerative diseases [127,128]. In fact, even if free radicals support the immune system, facilitating cell signalling and playing a crucial role in apoptosis, they can damage nucleic acids, proteins, carbohydrates and lipids, leading to several diseases [129]. A wide number of natural antioxidants have been found, such as carotenoids, vitamin E, A, C, phenols and other different compounds [45], and their consumption, as food supplements, results in reduced risk of many diseases.

Some Sardinian endemic species exerted antioxidant activity. The essential oil of *A. caerulescens* subsp. *densiflora* was found to be able to protect unsaturated lipids in β-carotene-linoleate system, while it showed only a weak antioxidant activity in the 1,1-diphenyl-2-picrylhydrazyl (DPPH) test [98]. It was also promising as a ClO^−^ scavenger [98].

The antioxidant activity of methanol, petroleum ether and ethyl acetate extracts obtained from the aerial parts of *B. morisiana*, and three isolated compounds (erybraedin C, bitucarpin A and plicatin B), was investigated in several models of lipid oxidation [130]. Both in linoleic acid and iron-mediated oxidation tests, the ethyl acetate and petroleum ether extracts were the most effective, with respect to the methanol one, which although less effective, still showed an interesting preventive activity [130]. The same extracts also showed a significant inhibition of the cholesterol autoxidation [130]. The ethyl acetate and petroleum ether extracts did not exert cytotoxic activity at antioxidant concentrations < 10 mg/mL, and the methanol one was nontoxic at all concentrations tested. Erybraedin C and plicatin B were capable of protecting linoleic acid against a free radical attack, in a manner superior to that exerted by α-tocopherol, highliting a noteworthy antioxidant activity. Erybraedin C was also active against cholesterol autoxidation and oxidative damage induced in VERO (monkey kidney ephitelial) cells by FeCl_3_ [130]. Instead, bitucarpin A, its deprenyl and dimethyl derivative, was not active, suggesting that two hydroxyl groups in the aromatic ring with prenyl groups in the *ortho* position are essential for the antioxidant activity [130].

The seeds’ oil obtained from *E. pithyusa* subsp. *cupanii* and *E. semiperfoliata* exhibited an interesting antioxidant activity in DPPH and β-carotene bleaching tests. This activity was significantly correlated with the tocopherol content [131]. Interestingly, *E. pithyusa* subsp. *cupanii*, which showed a consistently lower content of α-tocopherol than *E. semiperfoliata*, revealed a higher scavenging activity against DPPH radical, revealing the more important role of β- and γ-tocopherol in scavenging activity in this test.

The presence of flavonoidic compounds, such as luteolin, genistein and 6-hydroxy-genistein in acetonic and ethanolic extracts of aerial parts from *Genista cadasonensis*, could explain the significant antioxidant activity exhibited in DPPH and 2,2′-azino-bis-3-ethylbenzthiazoline-6-sulphonic acid (ABTS) tests [132].

Methanol extract of *H. italicum* subsp. *thyrrenicum* possesses a good antioxidant activity, observed in DPPH and β-carotene bleaching tests [54]. This plant is characterized by various phenolics compounds, such as arzanol and helipirone, which exert a strong protective effect against linoleic acid and cholesterol oxidative degradation. In particular, arzanol caused a concentration-dependent reduction of DPPH similar to the well-known antioxidants such as L-cysteine or ascorbic acid [133] and, at nontoxic concentrations, exerted antioxidant activity also in a cultured cells model [134]. Further studies confirmed its powerful protective effect in lipid peroxidation systems [135]. Arzanol was able to prevent the oxidative damage to human low-density lipoprotein (LDL) and cell membranes [135]. Indeed, pre-treatment with arzanol significantly inhibited the increase of oxidative products, preserving lipoproteins from oxidative damage [135]. Furthermore, it reduced the formation of oxidative products from the degradation of unsaturated fatty acids and cholesterol, exerting, at non-cytotoxic concentrations, a remarkable protection on tert-butyl hydroperoxide (TBH)-induced oxidative damage in Vero and Caco-2 [135]. Interestingly, arzanol accumulates in Caco-2 epithelial cells, and a high rate of diffusion through intestinal cells layers, has been evidenced, suggesting that its antioxidant activity could depend on its bioavalability [135]. Recent studies have highlighted that both the hydroxylated aromatic group and the α-pyrone moiety are essential in the peroxyl radical scavenging properties, since the methylation of the pyrone moiety is detrimental for antioxidant activity [101].

*H. hircinum* subsp. *hircinum* and *H. scruglii* extracts exhibited a rather strong antioxidant activity in ABTS, DPPH, FRAP-ferrozine (ferric reducing/antioxidant power) and the β-carotene bleaching (BCB) tests [136,137]. This activity could be attributable to their phenolic components, since a significant linear correlation between this value and the antioxidant activity was observed [136,137].

The supercritical CO_2_ extract from *S. desoleana* aerial parts exerted, at increasing concentrations, antioxidant activity against H_2_O_2_-induced oxidative stress, on normal (HUVEC) and transformed (ECV304) human endothelial cells, failing to interfere with cell viability [138]. This property can be partially due to the presence of main constituents, especially sclareol, endowed with antioxidative actions [139].

Methanol and n-hexane extracts obtained from aerial parts of *Santolina corsica* revealed an interesting antioxidant activity determined by β-carotene bleaching, Ferric Reducing Activity Power (FRAP), DPPH and ABTS tests. Generally, the methanol extract was more effective than the n-hexane one [110].

The ethanol extract of *S. glutinosa* was screened by a wide number of assays, revealing a good scavenging activity attributable to polyphenols contents [118].

Methanol extracts from *Vinca difformis* subsp. *sardoa* were investigated through DPPH and ABTS assays [140]. Leaves possessed the highest activities, with respect to flowers and roots, probably because of the presence of quinic acid, chlorogenic acid, caffeoylquinic acid isomer and robinin, polyphenols widely recognized for their antioxidant properties [141,142,143].

#### 3.2.5. Anti-Inflammatory Activity

Inflammation is a natural defense mechanism activated by the immune system in response to different chemical, physical or biological factors. However, prolonged and chronic inflammation could be damaging and is a crucial risk factor for heart diseases, atherosclerosis, arthritis, neurodegenerative diseases, metabolic disorders and cancer [144]. Numerous inflammatory mediators such as cytokines, chemokines, eicosanoids and the inflammation-modulating transcription nuclear factor kβ (NF-*k*B) are synthetized and secreted during inflammatory responses [145]. Therefore, inhibiting their release or ameliorating the dysregulation of pro-inflammatory and anti-inflammatory cytokines, such as Interleukin-1 β (IL-1β), Interleukin-6 (IL-6), Tumor necrosis factor α (TNF-α) and Interleukin-10 (IL-10), is a potential strategy for the treatment of inflammation. Current therapies are mainly based on steroidal and non-steroidal drugs and glucocorticoids that, although particularly effective, are not devoid of side effects that limit their use over long periods [144,146]. Hence, discovering new anti-inflammatory agents that are potentially non-toxic is urgently required. Among the Sardinian endemic species, five taxa belonging to Asteraceae (*Helichrysum italicum* subsp. *tyrrhenicum*, *Plagius flosculosus*, *Santolina corsica* and *Santolina insularis*) and Lamiaceae (*Salvia desoleana*) exhibited antinflammatory activity.

Arzanol isolated from *H. italicum* subsp. *tyrrhenicum*, and previously cited for other significant properties, showed powerful NF-*k*B-inhibiting action [83]. The NF-*k*B is a validated target for inflammation [147] since it is one of the main regulators of genes involved in the regulation of several factors implicated in inflammatory conditions. Arzanol also showed a potent inhibition of the production of IL-1β and TNFα, pro-inflammatory mediators in primary human monocytes [83]. Furthermore, it inhibited in vitro biosynthesis of eicosanoids, such as 5-lipoxygenase and cyclooxygenase (COX)-1, and also reduced in vivo prostaglandin E2 (PGE2) levels [133].

Ethyl acetate extract and a polyacetylene spiroketal isolated from *P. flosculosus* inhibited the induction of NF-*k*B activity. This compound, known as tonghaosu and also found in herb chamomile [148], inhibited the phosphorylation and proteasomal degradation of the IkB protein, preventing the nuclear import and DNA binding of NF-*k*B and interfered with the lipopolysaccharide (LPS)-induced production of IL-1, IL-6, TNF and prostaglandin PGE2 in primary human monocytes [149].

Essential oil from *S. desoleana* exhibited a good level of inhibition of carrageenan-induced edema, on an in vivo model of anti-inflammatory activity, compared to indomethacin [150].

The essential oil of *S. corsica*, which was characterized by three chemotypes (artemisia ketone, myrcene and β-phellandrene), exhibited anti-inflammatory activity on the bronchial tract [151]. In fact, when human cells resulted from bronchoalveolar lavage, rich in macrophage-histiocytes, were incubated with the essential oil, the histiocytes restored their ability to phagocytize more material. The addition of the essential oil also produced an evident decrease of granulocytes [151]. The n-hexane extract from the same plant decreased nitric oxide (NO) production and COX-2 levels, showing anti-inflammatory activity [110].

Several flavonoids isolated from *S. insularis* showed topical anti-inflammatory activity inhibiting croton oil-induced ear edema in mice [152]. Luteolin, according to the literature [153], was the most active compound with a higher reduction of edema with respect to the positive control. A recent study demonstrated that pre-treatment with the essential oil of this plant decreased NO production induced by LPS and the expression of iNOS and COX-2, two key enzymes upregulated during fungal infections [71]. Since the oil has no scavenging activity towards NO, it could act via NF-*k*B pathway modulation.

#### 3.2.6. Anti-Aging Activity

Skin aging is a natural consequence of structural changes in skin structure and elasticity. It is genetically determined but can also be considerably influenced by environmental factors and lifestyle components [154]. Particularly, the exposure to ultraviolet (UV) light accelerates the ageing of skin, also increasing the risk of dermatological disorders [155]. UV radiation causes the upregulation of matrix metalloproteinases-activating enzymes, such as elastase and tyrosinase, responsible for the deterioration of the dermal extracellular matrix components, thus involved in the skin ageing process as well as in many dermatological diseases [156,157]. Therefore, compounds acting as their inhibitors could be useful as anti-wrinkles and skin-whitening agents. Several plant compounds, endowed with antioxidant properties, have been reported to modulate the activity of these enzymes [158]. Among Sardinian endemic species, *Hypericum hircinum* subsp. *hircinum* and *Limonium morisianum* were able to inhibit both enzymes’ activities, while *Hypericum scruglii* exhibited a selective activity on elastase, suggesting its potential use as an ingredient for anti-wrinkles cosmetics [159]. A positive correlation was established among the enzymatic inhibition and the total phenolic and flavonoid contents [159]. *H. hircinum* subsp. *hircinum* and its pure compounds quercetin, caffeoylquinic acids and 5,7,3′,5′-tetrahydroxyflavanone, as well as bulbs extract of *P. illyricum*, were also capable of inhibiting collagenase, another enzyme responsible for the degradation of all components of the extracellular matrix, such as collagen and elastin [62,136].

#### 3.2.7. Estrogenic/Antiestrogenic Activity

Phytoestrogens are a group of natural compounds, which have estrogen-like activity [160], with health benefits, such as a lowered risk of heart disease, breast and other hormone-dependent tumors, menopausal symptoms and osteoporosis. Three main categories of phytoestrogens, namely isoflavones, lignans and coumestans, are present in numerous vegetables (most notably soy), fruits and medicinal plants [161], and they are marketed as a natural alternative to estrogen replacement therapy. Nevertheless, phytoestrogens can potentially cause negative health effects, since they could have anti-estrogenic properties, acting as endocrine disruptors [162]. Their activities are due to the structural similarity with 17-β-estradiol, the primary female sex hormone that promotes the interaction with the two main α and β estrogen receptors. Consequently, phytoestrogens can act as agonists, partial agonists and antagonists [160,163]. Some isoflavones and flavones isolated from the aerial parts of *Genista morisii* and *Genista ephedroides* were in vitro evaluated for their estrogenic [164] and anti-estrogenic activities [165] by a yeast-based estrogen receptor assay. The licoflavone C and the isoflavone genistein showed the highest estrogenic activity detected by β-galactosidase activity induction. The affinity between the licoflavone C and the estrogen receptor α was probably due to the presence of an isoprenyl substituent at ^8^C [166]. Interestingly, among the active isoflavones (daidzein, genistein, isoprunetin), the estrogenic activity was influenced by glucosilation, since, in general, aglycones were more active than the corresponding glucosides [164]. According to the observation that compounds possessing weak estrogenic activities may exert more appreciable inhibitory action, luteolin, isolated from *G. morisii*, inhibited the activity of β-galactosidase mediated by 17-β-estradiol, exhibiting anti-estrogenic properties [165]. Differently, licoflavone C acted as an estradiol inhibitor within a concentration range but was estrogenic at higher concentrations. Isoprunetin and isoprunetin 7-glc, that exerted a relatively weak estrogenic action, possessed both agonistic and antagonist activity [165].

#### 3.2.8. Antidiabetic Activity

Diabetes is one of the largest worldwide health emergencies since it currently affects hundreds of millions of people, with a rising trend [167]. It is due, in 85–90% of total cases, to the poor or no response of receptors to insulin (type 2 diabetes mellitus), that increase blood glucose levels. Alternatively, type 1 diabetes mellitus, more frequent in children and teenagers, is imputable to the lack or deficiency of insulin, depending on the inability of pancreatic cells to produce it [168]. Diabetes is frequently associated to several complications, most notably cardiovascular and metabolic disorders. One of the therapeutic approaches to treat diabetes consists of the inhibition of carbohydrate-hydrolyzing enzymes (such as α-amylase and α-glucosidase). The former is responsible for the breakdown of polysaccharides such as starch to monosaccharides [169], the latter breaks down carbohydrates into monosaccharide glucose, allowing its absorption by the intestine [168]. Acarbose, miglitol and voglibose, as well as other synthetic enzyme inhibitors, reduce the absorption rate of glucose, causing a reduction in postprandial glucose levels [170]. However, they are known to produce side effects; therefore, there is an urgent need to search for effective and safer enzyme inhibitors. Several plants have displayed effective inhibitory activity against carbohydrate-hydrolyzing enzymes [171,172,173].

Among Sardinian endemics, *H. hircinum* subsp. *hircinum*, *H. scruglii* and *Limonium contortirameum* exhibited antidiabetic properties. Both *Hypericum* taxa exerted a significant α-glucosidase inhibition [137], while *Limonium contortirameum* was able to inhibit both α-amylase and α-glucosidase enzymes [174]. Quercetin, identified in these species both as aglycon and glycoside forms, could be responsible, together with other metabolites, for the activity observed, since it is known to reduce blood glucose levels, improving plasma insulin levels [175]. *L. contortirameum* was also able to inhibit pancreatic triacylglycerol lipase [174], an enzyme important in triacylglycerol breakdown and widely used to determine the potential efficacy of natural products as obesity modulating agents [176]. According to the literature [177], both crude extract and gallic acid isolated from this plant exhibited significant anti-obesity effects.

#### 3.2.9. Other Activities

Along with the main biological activities described above, some species also exhibited other peculiar properties. *H. hircinum* subsp. *hircinum* also showed cardioprotective and monoamine oxidase (MAO) inhibition properties, confirming that it is one of the most studied Sardinian endemic species. Shah and coauthors [178] described the ability of crude extract obtained from its aerial parts to reduce the cardiotoxicity induced by doxorubicin in rats. This anthracycline glycoside antibiotic is an effective chemotherapeutic drug used for many types of cancer and its toxicity is well-known [179]. The mechanism responsible for doxorubicin-induced cardiotoxicity is the formation of reactive oxygen species (ROS), leading to oxidative stress [180]. Administration of *H. hircinum* subsp. *hircinum* in rats induced a decrease of the levels of lipid peroxidative value and marker enzymes, increased the levels of glutathione and superoxide dismutase and prevented the decrease in heart weight in the DOX-treated group [178]. These results may be attributable to its phenolic content and its strong antioxidant activity, as previously reported, suggesting the potential use of this plant as a cardioprotective agent. The positive effect of *H. scruglii* extract against fibromyalgia could also be attributed to the antioxidant activity [181], since the oxidative stress plays a significant role in the pathophysiology of this syndrome. Moreover, the methanol extract and quercetin isolated from *H. hircinum* subsp. *hircinum* showed inhibition of monoamine oxidase (MAO) [182], suggesting a potential role in the treatment of neurological disorders. In fact, MAO regulation seems to play a central role in several neurological disorders, such as clinical depression and Parkinson’s Disease [183]. Quercetin was also tested to determine the activity towards MAO-A and MAO-B, revealing a selective inhibitory activity against MAO-A [182].

In addition to the previously described anti-inflammatory properties, *S. desoleana* essential oil exherted analgesic properties in an animal model [150]. Interestingly, this oil was able to permeate the in vitro porcine buccal mucosa in Franz cells, making possible its use in different formulations, in the stomatological field for its antimicrobial and anti-inflammatory properties [184].

A new galanthamine-type compound, 11α-hydroxy-O-methylleucotamine, extracted from the leaves of *P. illyricum*, has shown a good in vitro acetylcholinesterase inhibitory activity, making this plant a potential source of compounds to be employed for the treatment of Alzheimer diseases [185]. Interestingly, leaves (not bulbs) were particulary rich in the active alkaloids; thus, the safeguard of this endemic species could be guaranteed.

The dichloromethane extract and xanthomicrol, the latter a flavone obtained from aerial parts of *S. glutinosa*, exhibited binding affinity for *μ* and *δ* opioid receptors. It has also been shown, in an animal model, that pretreatment with xanthomicrol, administered intraperitoneally in mice, significantly inhibited the antinociception induced by morphine in a dose-dependent manner, suggesting an antagonistic effect on *μ* opioid receptors [186]. These results acquire considerable value since opioid antagonists are effective in the control of alcohol dependence and several mental pathologies [187,188].

### 3.3. Phytoconstituents

Forty-six endemic plants have been subjected to phytochemical studies and a large variety of phytoconstituents, such as alkaloids, terpenoids, phenolic compounds, saponins, volatile constituents and fatty acids, has been described. A complete list of secondary metabolites identified to date from Sardinian endemic flora is reported in Table 3.

The most studied plant is *Helichrysum italicum* subsp. *tyrrhenicum*, with 15 articles highlighting the existence of significant differences in both qualitative and quantitative analyses, especially on essential oils. This high variability, observable also in *Artemisia caerulescens* subsp. *densiflora*, *Santolina corsica*, *Santolina insularis* and *Thymus herba-barona* subsp. *herba-barona*, supported the existence of various chemotypes into these species [46,52,53,97,98,151,189,190,191,192,193]. This chemodiversity could be attributable to genetic and environmental factors [194]. In fact, it is well known that the different geographic localities, seasons, harvest periods, soil properties and climatic conditions strongly affect the phytochemical pattern of plants, especially essential oils. In the case of *H. italicum* subsp. *tyrrhenicum*, the relationship between climatic variables and chemical compounds of the volatile fraction was recently described, revealing that nerolidol is mostly positively correlated to mean winter temperature, while italicene, bergamotene, nerol and curcumene are positively influenced by spring and summer precipitation [193]. The same authors also established a correlation between essential oils profiles and the altitude, revealing that the content of italicene, γ-curcumene, ar-curcumene, nerol and geraniol is positively influenced by the altitude, while limonene, caryophyllene, nerolidol and cis-β-guaiene showed a negative correlation [193].

In addition to genetic and environmental factors, the extraction methods were also shown to induce differences in chemical types or contents in the extracts [45]. Indeed, the yield of some compounds extracted by supercritical fluid extraction (SFE) from *Plagius flosculosus*, *Salvia desoleana*, *Santolina. insularis*, *Seseli praecox* and *Tanacetum audibertii* was often different in comparison to that obtained by the hydrodistillation method [64,70,76,138,195].

It is noteworthy that, in some cases, the presence or absence of particular metabolites within congeneric species acquired a chemotaxonomic value. For example, in *Genista* ssp., despite the presence of aliphatic and unsaturated aldehydes, which represented a common feature, some volatile compounds that were pointed out as a target of single species have been detected: n-heptanal for *G. arbusensis*, and (E, Z)-2,6-nonadienal for *G. corsica* [196].

**Figure 4:**
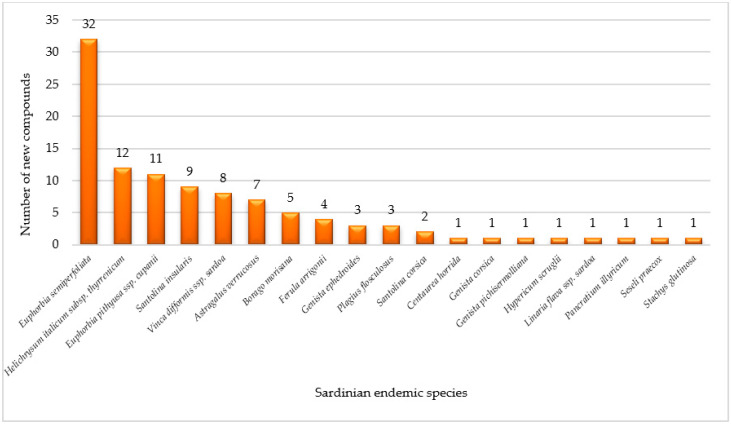
Number of compounds isolated for the first time from Sardinian endemic species (*n* = 104).

Remarkably, bioprospecting of Sardinian endemic taxa has yielded, along with a high number of known compounds, one-hundred and four previously undescribed metabolites. Out of the new compounds, some are endowed with interesting biological properties [48,81,82,86,105,185]. In particular, the highest number of new metabolites has been detected from *E. semiperfoliata*, *E. pithyusa* ssp. *cupanii* and *H. italicum* subsp. *thyrenicum* [57,81,82,197,198,199,200], as shown in Figure 4. The identification and isolation of original metabolites is worth noting, since they could provide new scaffolds for drug design.

**Table 3:** Phytoconstituents isolated from Sardinian endemic species.

Taxon	Type of Extract	Plant Organ	Main Constituents	Voucher Specimen	References
*Artemisia caerulescens* subsp. *densiflora*	Essential oil	Flowers	Camphor, isoborneol, terpinen-4-ol, camphene	N.A.	[191]
	Essential oil	Leaves	α-thujone, terpinen-4-ol, camphor	N.A.	[191]
	Essential oil	Aerial parts	Terpinen-4-ol, p-cymene, ʏ-terpinene, 1,8-cineole, α-terpineol	Herbarium SASSA 736	[46]
	Essential oil	Aerial parts	Davana ethers, cis-Sabinene hydrate, terpinen-4-ol, (E)-nerolidol, β-Oplopenone	Herbarium CAG 736	[98]
	Ethanol extract	Aerial parts	α/β dihydroartemisinin, artemisinin, artemisic acid, absinthin, phytol, stigmasterol, 3-O-caffeoylquinic acid, caffeic acid, 3-O-caffeoyl-5-O-feruloylquinic acid, isofraxidin, apigenin, quercetin 3-methyl ether, eupalitin, luteolin-7-O-methyl ether, axillarin and cirsimaritin	Herbarium CAG 736	[201]
*Astragalus verrucosus*	Ethyl acetate and n-buthanol extracts	Aerial parts	Saponins: astraverrucins I **, II **, III **, IV **, V **, VI **; D-pinitol	URB-96/357	[202,203,204]
	Ethyl acetate and n-buthanol extracts	Aerial parts	Saponins: astraverrucin VII **, cycloaraloside D (peregrinoside II) and cycloaraloside C (astrailienin A)	URB-96/357	[204]
	Chloroform, ethyl acetate and n-buthanol extracts	Aerial parts	Flavonoids: Daidzein, genistein, apigenin, apigenin 7-glucoside, nicotiflorin, rutin, apigenin 7-O-β-D-(6-Op-coumaroyl)glucoside, kaempferol 3-O-robinobioside, quercetin 3-O-robinobioside, daidzin, ononin, calycosin, psudobaptigenin, genistin, pratensein	URB-96/357	[204]
	Chloroform, ethyl acetate and n-buthanol extracts	Aerial parts	Pterocarpan derivative: maackiain	URB-96/357	[204]
*Bituminaria morisiana*	Acetone extract	Aerial parts	Erybraedin C, bitucarpin A **; 8-prenyldaidzein, plicatin B	Herbarium CAG 391/B	[205]
	Petroleum ether, ethyl acetate and methanol extracts	Aerial parts	Erybraedin C, plicatin B, and bitucarpin A	Herbarium CAG 391/B	[130]
	Petroleum ether and ethyl acetate extracts	Leaves	Erybraedin C, bitucarpin A, 3,9-dihydroxy-4-(3,3-dimethyallyl)[6aR,11aR]-pterocarpan **, 3-hydroxy-4-(3,3-dimethylallyl)-4*″*,5*″*-dehydropyrano[8,9:2*″*,3*″*][6aR,11aR]-pterocarpan **, 4*′*,5*″*-dihydroxy-6*″*-methoxy-4*″*,4*″*-dimethyl-4*″*,5*″*-dihydro-6*″*H-pyrano[7,8:2*″*,3*″*]-isoflavone **, daidzein, 8-prenyldaidzein, bidwillon C, coumestrol, pseudoisopsoralen	Herbarium of Dipartimento Farmaco Chimico Tecnologico, University of Cagliari (No. 0201)	[102]
	Ethyl acetate extract	Seeds	3-hydroxy-4-(3*′*-methyl-2*′*-butenyl)-furo[2*′*,3*′*:8,9][6aR,11aR]pterocarpan (morisianine) **, erybraedin C, psoralen, angelicin	Herbarium of Dipartimento Farmaco Chimico Tecnologico, University of Cagliari (No. 0201)	[103]
*Borago morisiana*	Fatty acid methyl esters (FAME)	Seeds	γ-linoleic and stearidonic acids	HUAL 25639	[206]
				HUAL 25965	
*Borago pygmaea*	Fatty acid methyl esters (FAME)	Seeds	γ-linoleic and stearidonic acids	HUAL 25608	[206]
*Centaurea horrida*	Methanol extract	Aerial parts	Flavonoid glycoside: horridin **	URB-3214/97	[207]
	N-hexane, chloroform, chloroform-methanol and methanol extracts	Aerial parts	Lupeol, betulin, apigenin, 5-caffeoylquinic acid, β-sitosterol 3-O-β-D-glucopyranoside, rutin, 3-caffeoylquinic acid, apigenin 3-O-β-D-glucopyranouronide, apigenin 8-C-α-1-arabinopyranoside, apigenin 6-C-α-L-arabinopyranoside, protocatechuic acid, scutellarein 7-O-β-D-glucopyranoside, quercetin 3-O-α-L-rhamnopyranoside, apigenin 7-O-β-D-glucopyranoside, kaempferol 3-O-β-D-glucopyranoside, kaempferol 3-O-α-L-rhamnopyranoside, horridin, cis- and trans-3,5-dicaffeoyl quinic acids, vitexin, isovitexin, orientin, shaftoside, apigenin 6,8-di-C-β-D-glucopyranoside and 4-caffeoylquinic acid	Herbarium Horti Botanici Pisani 03/7219	[208]
*Cymbalaria muelleri*	Ethanol extract	Aerial parts	Iridoid glycosides: antirrhinoside, antirrhide, macfadienoside, 7-β -hydroxy 8-harpagide	N.A.	[209]
*Euphorbia hyberna* subsp. *insularis*	Acetone extract	Aerial parts	Jatrophane diterpenoids	N.A.	[210]
*Euphorbia pithyusa* subsp. *cupanii*	Acetone extract	Aerial parts	Lathyrol-3-phenylacetate-5,15-diacetate **, premyrsinol-3-propanoate-5-isobutyrate-7,13,17-triacetate **, premyrsinol-3-propanoate-5-isobutyrate-7,13-diacetate-17-nicotinate **, premyrsinol-3-propanoate-5(R-methyl)butyrate-7,13-diacetate-17-isobutyrate **, premyrsinol-3-propanoate-5,17-diisobutyrate-7,13-diacetate **, premyrsinol-3,17-dipropanoate-5-isobutyrate-7,13-diacetate **, premyrsinol-3-propanoate-5-benzoate-7,13,17-triacetate **, premyrsinol-3-propanoate-5-isobutyrate-7,13,17-triaacetate **, 4,12,20-trideoxyphorbol-13-(2,3-dimethyl)butyrate **, 4,12-dideoxyphorbol-13-(2,3-dimethyl)butyrate **, 4,12-dideoxyphorbol-13-(2,3-dimethyl)butyrate-20-acetate **	Herbarium CAG 1212	[197]
	Fatty acid methyl esters (FAME)	Seeds	palmitic acid, stearic acid, oleic acid, linoleic acid, linolenic acid	Herbarium CAG 1212	[131]
	Unsaponifiable fraction	Seeds	hydrocarbons, fatty alcohols, campesterol, β-sitosterol, Δ^5^-avenasterol, cycloartanol, 24-methylen-cycloartenol	Herbarium CAG 1212	[131]
	Tocopherols	Seeds	α-tocopherol, β-tocopherol, γ-tocopherol, δ-tocopherol	Herbarium CAG 1212	[131]
*Euphorbia semiperfoliata*	Ethyl acetate extract *	Whole plant	Eleven Jatrophane esters **, three 4-deoxyphorbol esters **		[81,82,199]
	Acetone extract	Aerial parts	Scopoletin, helioscopinolides A and B, an abietanolide **, 13 jatrophane polyesters **, two 4-deoxyphorbol diesters **, 2 epimeric diterpenes **	Herbarium CAG 1217	[198]
	Acetone extract	Aerial parts	Jatrophane polyesters	N.A.	[104]
	Fatty acid methyl esters (FAME)	Seeds	myristic acid, palmitic acid, stearic acid, oleic acid, linoleic acid, linolenic acid, arachidic acid, behenic acid	Herbarium CAG 1217	[131]
	Unsaponifiable fraction	Seeds	hydrocarbons, fatty alcohols, cholesterol, campesterol, β-Sitosterol, lanosterol isomer, lanosterol, β-amyrin, cycloartanol, 24-Methylen-cycloartenol	Herbarium CAG 1217	[131]
	Tocopherols	Seeds	α-tocopherol, β-tocopherol, γ-tocopherol, δ-tocopherol	Herbarium CAG 1217	[131]
*Euphrasia nana*	Ethanol extract of aerial parts	Aerial parts	Iridoid glucosides: aucubin, catalpol, mussaenosidic acid and melampyroside	N.A.	[211]
*Ferula arrigonii*	N.A.	N.A.	7,11-dehydrogrilactone * (jalcaguaianolide derivative)	N.A.	[212]
	Acetone extract	Roots	Coumarin derivatives: colladonin, colladin, badrakemone, umbelliprenin, kataravicinol, isosamarkandin angelate; daucane esters: ferutidin, lapiferin, 2α-Hydroxiferutidin **, 2-oxoferutidin **, latifolone	N.A.	[106]
	Acetone extract	Fruits	Lapiferin, ferutidin, Jaeskeanadiol veratrate, webbiol angelate, 10α-hydroxyferutidin, 10-deangeloylpallinin **, laserin	N.A.	[106]
*Galium corsicum*	Ethanol extract	Aerial parts	Iridoids: asperuloside, monotropein, asperulosidic acid, scandoside, loganic acid; coumarin	Herbarium CAG 652	[213]
*Galium glaucophyllum*	Ethanol extract	Aerial parts	Iridoids: asperuloside, monotropein, asperulosidic acid, deacetyl-asperuloside, geniposidic acid, loganin and loganic acid	Herbarium CAG 654	[213]
*Galium schmidii*	Ethanol extract	Aerial parts	Iridoids: asperuloside, monotropein, geniposidic acid, loganin and 10-hydroxy-loganin	Herbarium CAG 654/a	[213]
*Genista arbusensis*	Essential oil	Flowers	Heptanal, 1-octen-3-ol, (E,Z)-2,6-nonadienal, (E)-2-(2-pentenyl)-furan, 2-penthylfuran, (E)-2-hexenal	GA120503NE	[196]
*Genista bocchierii*	Essential oil	Flowers	Caryophyllene oxide, 1-octen-3-ol, heptanal, β-cariophyllene, n-pentadecane, (E,Z)-2,6-nonadienal	GB240504PU	[196]
*Genista cadasonensis*	Essential oil	Flowers	1-octen-3-ol, (E)-2-(2-pentenyl)-furan, linalool, 2-penthylfuran, (E,Z)-2,6-nonadienal	GD290403CS	[196]
	Ethanol extract	Aerial parts	Flavonoids: luteolin, genistein and 6-hydroxy-genistein	Herbarium CAG 290/A	[132]
	Ethanol extract	Fruits	Pinitol, 3-methoxy-chiro-inositol	Herbarium CAG 290/A	[132]
*Genista corsica*	Essential oil	Flowers	(E,Z)-2,6-nonadienal, 1-octen-3-ol, E-β-farnesane, (E)-2-hexenal, (E)-2-nonenal, mesitylene	GC230402CA	[196]
	Ethyl acetate extract	Leaves	Daidzein and luteolin	N.A.	[214]
	N.A.	N.A.	Quinolizidine alkaloids: anagyrine, cytisine, N-methylcytisine, lupanine, retamine and sparteine	N.A.	[215]
	N-hexane, choloroform and methanol extracts	Aerial parts	Dihydroisoderrondiol **, daidzein, luteolin, luteolin 4′-O-β-glucoside, luteolin 7-O-β-glucoside, isoprunetin, isoderrone, ficuisoflavone, taxifolin, 5-methoxytaxifolin, sucrose, D-pinitol	URB-1742/96	[216]
*Genista ephedroides*	N-hexane, choloroform and methanol extracts	Aerial parts	Hydroxyalpinumisoflavone **, ephedroidin **, genisteon **, genistein, isoprunetin, wighteone, laburnetin, alpinumisoflavone, genistin, genistein 8-C-glucoside, apigenin, isokaempferide, licoflavone C, pinitol	URB-167/94	[217]
	N.A.	N.A.	Alkaloids: sparteine, lupanine, anagyrine, cytisine, N-methylcytisine, retamine	N.A.	[215]
	N-hexane, choloroform and methanol extracts	Aerial parts	Alkaloids: lupanine, anagyrine, 17-oxoretamine, 12-α-hydroxylupanine, retamine	URB-167/94	[218]
	N-hexane, choloroform and methanol extracts	Aerial parts	Licoflavone C	N.A.	[125,164,165]
*Genista morisii*	Essential oil	Flowers	*n*-pentacosane, (E)-2-(2-pentenyl)-furan, (E)-2-hexenal, 2-penthylfuran, (E,Z)-2,6-nonadienal, E-*β*-farnesane	GM080403SA	[196]
	Hydrolyzed extracts	Leaves	Flavonoids: daidzein, genistein, isoprunetin, luteolin	N.A.	[214]
	N-hexane, choloroform and methanol extracts	Aerial parts	Flavonoids: Genistein, daidzein, isoprunetin, eriodictyol, genistein-7-O-β-D-glucopyranoside, isoprunetin 7-O-β-D-glucopyranoside, vitexin, orientin, luteolin, luteolin 7-O-β-D-glucopyranoside, isoprunetin 7,4*′* -di-O-β-D-glucopyranoside, genistein 7,4*′*-di-O-β-D-glucopyranoside	Herbarium SASSA 287	[219]
	N-hexane, choloroform and methanol extracts	Aerial parts	Flavonoids: daidzein, genistein, genistein 7-O-β-D-glucopyranoside, isoprunetin, isoprunetin 7-O-β-D-glucopyranoside, isoprunetin 4*′*,7-di-O-β-D-glucopyranoside, luteolin, luteolin 7-O-β-D-glucopyranoside, luteolin 4*′*-O-β-D-glucopyranoside	N.A.	[164,165]
*Genista pichisermolliana*	Essential oil	Flowers	β-myrcene, nerol, (E)-2-(2-pentenyl)-furan, γ-curcumene, linalool, nonanal, neryl acetate	GP130603LA	[196]
	Petroleum ether, chloroform and methanol extracts	Aerial parts	Alpinumisoflavone 4*′*-O-glucopyranoside **, daidzein, genistein, genistein 7-O-β-glucopyranoside, biochanin A 7-O-β-glucopyranoside, genistein 4*′*,7-di-O-β-glucopyranoside, genistein 8-C-β-glucopyranoside, orobol 8-C-β-glucopyranoside, 3*′*-O-methylorobol 8-C-β-glucopyranoside, rutin, quercetin 3-O-robinobioside, isorhamnetin 3-O-β-glucopyranoside, isorhamnetin 3-O-β-galattopyranoside, isorhamnetin 3-O-robinobioside, apigenin, luteolin 7-O-β-glucopyranoside, eriodictiol, aromadendrin 7-O-β-glucopyranoside, maackiain, 4-methoxymaackiain, p-coumaric methylester and D-pinitol	Herbarium CAG 290/b	[220]
*Genista sulcitana*	Essential oil	Flowers	1-octen-3-ol, (E)-2-hexenal, β-myrcene, (E,Z)-2,6-nonadienal, (E)-2-(2-pentenyl)-furan, 3-octanol	GS090503MV	[196]
	Methanol extract	Aerial parts	luteolin, 7-O-glucoside, genistein 7-O-glucoside, genistein 8-C-glucoside, p-coumaric acid	N.A.	[221]
*Glechoma sardoa*	Essential oil	Aerial parts	β-elemene, δ-elemene, γ-elemene, isogermafurene	URB-GS 165	[51]
	Essential oil	Aerial parts	germacrene D, β-elemene, isogermafurene, δ-elemene, β-phellandrene, elemol, γ-elemene, δ-elemene	N.A.	[222]
*Helichrysum italicum* subsp. *tyrrhenicum*	Essential oil	Aerial parts	Neryl acetate, nerol, neryl propionate, linalool, rosifoliol, γ -curcumene, γ-cadinene, δ-cadinene	Herbarium SASSA 729 [53,192,193]	[52,53,97,192,193,223,224,225,226]
				Herbarium CAG 729 [97]	
	Methanol extract	Aerial parts	α-terpinolene, trans-cariophyllene and neryl acetate)	N.A.	[54]
	Acetone extract	Aerial parts	Arzanol (phloroglucinol α-pyrone), oleyl ω-hydroxylinalol, helipyrone, tremetones, mycropyrone	Herbarium CAG 729	[83]
	Acetone extract	Aerial parts	Arzanol, methylarzanol, helipyrone, micropyrone, rosifoliol, 10-hydroxytremetone, acetoxytremetone, acetoxyhydroxytremetone	N.A.	[101,134,135]
	Acetone extract	Aerial parts	Arzanol	N.A.	[133]
	Acetone extract	Aerial parts	Arzanol, ursolic acid, neryl acetate, ω-oleoyloxylinalol, two O-geranylated isomeric coumarates, helipyrone, oleoylbitalin A **, nonanoylbitalin A **, propanoylbitalin A **, isocaproylbitalin A **, six angeloylated lipids (santinols) **, micropyrone, heliarzanol **	Herbarium CAG 729/10	[57]
	Acetone extract	Aerial parts	Micropyrone, arzanol, helipyrone, two acylic derivatives of bitalin A, gnaphaliol, caffeic acid, ursolic acid, 7-O-β-(D-glucopyranosyl)-5-methoxy-1(3H)-isobenzofuranone, gnaphaliol-9-O-β-D-glucopyranoside, gnaphaliol-3-O-β-D-glucopyranoside, 6-O-β-(D-glucopyranosyl)-4-methoxy-1(3H)-benzofuranone **	Herbarium CAG 729	[200]
*Helichrysum saxatile* subsp. *saxatile*	Essential oil	Flowers	α-pinene, limonene, 1,8-cineole, γ-curcumene. β-caryophyllene	HMGBH.e/9006.2015.009	[227]
*Hypericum hircinum* subsp. *hircinum*	Methanol extract	Leaves	Quercetin, eriodictyol, 1,6-dihydroxy-5,7-dimethoxyxanthone, (4R)-4-hydroxy-5,5-dimethyldihydrofuran-2-one, quercetin-3*′*-O-β-D-glucopyranoside and eriodictyol-7-O-β-D-glucopyranoside	Herbarium CAG 0201	[182]
	Hydroalcoholic and ethanol extracts	Aerial parts	Betulinic acid, shikimic acid, chlorogenic acid, quercetin, 5,7,3*′*,5*′*-tetrahydroxyflavanone, 5,7,3*′*,5*′*-tetrahydroxyflavanone-7-O-glucoside	Herbarium CAG 232	[84,136]
	Hydroalcoholic and ethanol extracts	Aerial parts	Shikimic acid, chlorogenic acid, quercetin, quercetin-7-O-glucoside, hypericin	Herbarium CAG 232	[137]
*Hypericum scruglii*	Hydro alcoholic and ethanol extracts	Aerial parts	Shikimic acid, chlorogenic acid, quercitrin, 3-geranyl-1-(2*′*-methylbutanoyl)-phloroglucinol, 3-geranyl-1-(2*′*-methylpropanoyl)-phloroglucinol, hyperoside, hypericin	Herbarium CAG 239/c	[137]
	Hydroalcoholic extract	Leaves	3-geranyl-1-(2*′*-methylbutanoyl)phloroglucinol, 3-geranyl-1-(2*′*-methylpropanoyl)phloroglucinol, 3-(13-hydroxygeranyl)-1-(2*′*-methylbutanoyl)phloroglucinol **, 1,3,5-benzentriol 2-[(2S,3R)-3-(3,4-dihydroxylphenyl)-2,3-dihydroxylpropyl], 3,4-dihydroxybenzoic acid and quercitrin	Herbarium CAG 239/c	[86]
*Limonium contortirameum*	Aqueous extract	Aerial parts	Quinic acid, gallic acid, (+)-catechin, quercetin 3-O-[6-(3 hydroxy-3-methyl-glutaroyl)B-D-galactoside, phlorizin, epigallocatechin, phloretin, epigallocatechin-3-gallate, 6*″*-galloylmyricetin-3-O-β-D-galactopyranoside, 6*″*-galloylmyricetin-3-O-β-D-glucoside, quercetin 3,4*′*-diglucoside, myricetin 3-O-β-D-glucopyranoside, myricetin 3-O-rhamnoside, myricetin 3-O-arabinopyranoside, quercetin 3-glucoside, myricetin 3-O-xylopyranoside, myricetin	Herbarium SASSA 909	[174]
*Limonium morisianum*	Methanol extract	Aerial parts	Myricetin, myricetin3-O-rutinoside, myricetin-3-O-(6*″*-O-galloyl)-β-D-galactopyranoside, (-)-epigallocatechin 3-O-gallate, tryptamine, ferulic and phloretic acids	Herbarium CAG 909/G	[88]
	Methanol extract	Aerial parts	Myricetin, (-)-epigallocatechin 3-O-gallate	Herbarium CAG 909/G	[90]
*Linaria flava* subsp. *sardoa*	N.A.	Aerial parts	Iridoid glucosides: 6*′*-O-acetylantirrhinoside **, antirrhinoside, 5-deoxyantirrhinoside, 5-glucosylantirrhinoside, antirrhide, linarioside, linaride, arcusangeloside	N.A.	[228]
*Mentha requienii* subsp. *requienii*	Essential oil	Aerial parts	Oxygenated monoterpenes: pulegone, isomenthone, isopulegone, limonene, linalool	Herbarium SASSA 1074 [47]	[47,229]
*Pancratium illyricum*	Methanol extract	Bulbs	Alkaloids: ungeremine, (−)-lycorine, (+)-vittatine	N.A.	[61]
	Dichloromethane extract	Bulbs	7,3*′*-dihydroxy-4*′*-methoxy-8-methyl flavan, 7,3*′*-dihydroxy-4*′*-methoxy flavan, p-hydroxyphenethyl trans-ferulate and sucrose	N.A.	[61]
	Methanol extract	Bulbs	Alkaloids: lycorine, 2-hydroxyhomolycorine, vittatine, galanthamine, sanguinine, habranthine	Herbarium CAG 1365	[185]
	Methanol extract	Leaves	Alkaloids: leucotamine, O-methylleucotamine, 11α-hydroxy-O-methylleucotamine **	Herbarium CAG 1365	[185]
	Methanol extract	Bulbs and Leaves	Alkaloids: galanthamine, sanguinine, vittatine, habranthine, lycorine, leucotamine, O-methylleucotamine, 2-hydroxyhomolycorine	Herbarium CAG 1365	[62]
*Plagius flosculosus*	Ethyl acetate extract	Aerial parts	3 polyacetylene spiroketals	N.A.	[149]
	Dichloromethane extract	Leaves	Flosculin A **, flosculin B **, flosculin C ** and other five diacetylenic spiroketal enol ethers	Herbarium CAG 0311	[109]
	Essential oil	Leaves	β-phellandrene, myrcene, iso-3-thujanol, (Z)-β-farnesene, β-pinene	Herbarium CAG 743	[64]
	Super critical Fluid Extract	Leaves	(Z)-β-farnesene, β-phellandrene, myrcene	Herbarium CAG 743	[64]
*Ptilostemon casabonae*	Hydroalcoholic extracts	Aerial parts	Quercetin, luteolin, kaempferol, apigenin and diosmetin O-glycosides, and caffeoylquinic acid derivatives	N.A.	[230]
*Salvia desoleana*	Essential oil	Leaves	Linalyl acetate, α-terpinil acetate, 1,8-cineole, α-terpineol, linalool, α-phellandrene, limonene, β-pinene, geranyl acetate, *trans*-linalool oxide, sabinene, geraniol, α-pinene	N.A.	[66,184]
	Essential oil	Leaves	Linalyl acetate, α-terpinil acetate, 1,8-cineole, linalool, germacrene D, α-terpineol, β-pinene, sabinene, sclareol, β-myrcene, limonene, α-pinene	N.A.	[65]
	Essential oil	Leaves	Germacrene D, α-terpinil acetate, sclareol, 1,8-cineole, linalool	N.A.	[138]
	Super critical fluid extract	Leaves	Sclareol, dotriacontano, vitamin E, nonacosane, terpenyl acetate, germacrene D, 1,8-cineole	N.A.	[138]
	Essential oil	Leaves	(Z)-β-Ocimene, α-Terpinyl acetate, 1,8-cineole, myrcene	N.A.	[231]
	Essential oil	Leaves	Samples from Gesturi: germacrene D, 1,8-cineole, α-terpinolene, α-terpinil acetate, bicyclogermacrene, α-pinene, sabinene, β-pinene, α-phellandrene. Samples from Luogosanto, Nuragus, Saccheddu, Villagrande: linalyl acetate, α-terpinil acetate, 1,8-cineole, germacrene D, linalool, β-myrcene, β-pinene	N.A.	[232]
	Essential oil	Aerial parts	Linalyl acetate, germacrene D, α-terpinil acetate, 1,8-cineole, linalool, α-terpineol	Herbarium CAG 1086	[91]
*Santolina corsica*	Essential oil	Aerial parts	Camphor, artemisia ketone, borneol, aromadendrene, muurolene	Herbarium CAG 732/A	[233]
	Essential oil *	Leaves	Acyclic sesquiterpenes aldehydes (3,9-dimethyl-6-isopropyl-2(*E*),7(*E*),9-decatrienal ** and 3,9-dimethyl-6-isopropyl-2(Z),7(E),9-decatrienal) **	N.A.	[234]
	Essential oil *	Aerial parts	Artemisia ketone, β-phellandrene, myrcene, santolina triene, 1,8-cineole, β-pinene, isolyratol	N.A.	[67]
	Essential oil *	Aerial parts	Myrcene, santolina triene, β-phellandrene, lyratol, β-pinene, sabinene	N.A.	[68,69]
	Essential oil	Aerial parts	Three chemotypes (artemisia ketone-β-phellandrene; myrcene; β-phellandrene-myrcene)	Herbarium SASSA 732	[151]
	Diethyl ether extract *	Roots	Monoterpenes (menthol); sesquiterpene hydrocarbons (β-sesquiphellandrene, α- and β-selinenes, β-elemene); terpenes and acetylene derivatives: (Z)-furylthienylbutenyne, (E)-furylthienylbutenyne, dammaradyenil acetate, friedeline, (Z)-acetoxymethylfurylthienylbutenyne, (E)-acetoxymethylfurylthienylbutenyne, 3-Epifriedelinol, dammaradienol, spiroketalenol ether, selin-11-en-4α-ol	N.A.	[235]
	Methanol extract *	Aerial parts	Kaempferol-3-O-glucoside, chlorogenic acid, rosmarinic acid, genistin, caffeic acid, ferulic acid, quercetin-3-O-glucoside, vanillic acid, protocatechuic acid, (-)-epicatechin, gallic acid, neochlorogenic acid	011151 and 011152	[110]
	N-hexane extract *	Aerial parts	Myrcene, palmitic acid methyl ester, palmitic acid ethyl ester, β-phellandrene, ar-curcumene, α-amyrin, β-amyrin, 1,8-cineole	011151 and 011152	[110]
*Santolina insularis*	Essential oil	Aerial parts	Artemisia ketone, 10-H-cyclopropyl-1,1,7-trimethyl-4-methylen-decahydro azulene, 1,8-cineole, camphene, bornyl acetate, borneol, α-pinene	Herbarium CAG 732 [233]	[93,233]
	Super critical fluid extract	Leaves	β-myrcene, *ar*-curcumene, β-phellandrene, trans-β-terpineol, spathulenol, γ-curcumene, 3-thujanol, 1,8-cineole, sabinene; waxes	N.A.	[70]
	Hydrodistillated essential oil	Leaves	β-myrcene, β-phellandrene, β-pinene, ar-curcumene, Spathulenol, trans-β-Terpineol	N.A.	[70]
	Essential oil	Aerial parts	Four chemotypes (artemisia ketone-β-phellandrene; cis-chrysanthemol-myrcene-β-pinene; β-phellandrene-myrcene; santolina triene)	Herbarium CAG 732 [74] Herbarium CAG 732/b [71] Herbarium SASSA 732 [73]	[71,73,74,189]
	Acetone extract	Aerial parts	Eudesmane sesquiterpenoids **, a trans-chrysanthemyl monoterpenoid **, two chrysanthemane monoterpenoids, eucamalol	Herbarium CAG 0707000	[236]
	Acetone extract	Aerial parts	11 polyoxygenated germacranes (four news **)	Herbarium CAG 732	[116]
	Methanol extract	Leaves	(E)-3-{6-[(E)-3-hydroxy-3-oxo-1-propenyl]-9-oxo-9H-xanthen-2-yl}-2-propenoic acid **, six flavonoids (hispidulin, nepetin, cirsimaritin, rhamnocitrin, luteolin and luteolin 7-O-β-D-glucopyranoside)	Herbarium of the Dipartimento Farmaco Chimico Tecnologico, University of Cagliari (No. 0310)	[152]
*Scrophularia trifoliata*	Ethanolic extract	Aerial parts	Iridoids: catalpol and aucubin	Herbarium CAG 27-3	[237]
*Seseli praecox*	Acetone extract	Aerial parts	Coumarins: anomalin, isopterixyn, 3*′*-angeloyl-(-)-cis-khellactone, bocconin **	N.A.	[238]
	Super critical fluid extract	Leaves	Differences between three sites (himachalol, sabinene, β-phellandrene; β-phellandrene, undecane, α-pinene; α-humulene, β-phellandrene, bicyclogermacrene)	N.A.	[195]
	Essential oil	Leaves	Differences between three sites (sabinene, β-phellandrene; β-phellandrene, α-pinene; α-humulene, β-phellandrene, bicyclogermacrene)	N.A.	[195]
*Stachys corsica*	Ethanol extract	Aerial parts	Flavonoid glycosides: isoscutellarein 7-*O*-(6*‴*-O-acetyl)-*β*-D-allopyranosyl-(1*‴*→2*″*)-*β*-D-glucopyranoside and isoscutellarein 4*′*-methyl ether 7-*O*-(6*‴*-*O*-acetyl)-*β*-D-allopyranosyl(1*‴*→2*″*)-*β*-D-glucopyranoside; iridoid glucosides: harpagide and acetylharpagide)	Herbarium CAG 29-1 [239]	[239,240]
*Stachys glutinosa*	Acetone and ethanol extracts	Aerial parts	Harpagide, 5-Allosyloxy-aucubin **, acetylharpagide, monomelittoside, melittoside, allobetonicoside	N.A.	[240,241]
	Essential oil *	Aerial parts	Terpinen-4-ol, α-pinene, α-terpineol, β-phellandrene and γ-terpinene, β-caryophyllene	N.A.	[242]
	Essential oil	Aerial parts	Differences between two sites (terpinen 4-ol, α-terpinyl acetate, trans-cadina-1(6),4-diene, α-terpineol; α-cedrene, α-terpineol, terpinen-4-ol, α-terpinyl acetate)	N.A.	[75]
	Dichloromethane extract	Aerial parts	Flavones: xanthomicrol, sideritoflavone, 8-methoxycirsilineol and eupatilin; roseostachenone and 3α,4α-epoxyroseostachenol **	Herbarium of the Department of Life and Environmental Science, Drug Sciences Section (No. 0425)	[186]
	Ethanol extract	Aerial parts	Melittoside, caffeoylquinic acid, 3-caffeoylquinic acid, chlorogenic acid, β-OH-forsythoside B, β-OH-acteoside, betonyoside E, hypolaetin-7-O-(2-allosyl)-glucopyranoside, forsythoside B, acteoside, isoscutellarein-7-O-[allosyl(1→2)]-glucopyranoside, isoacteoside, isoscutellarein-7-O-[6*‴*acetylallosyl-(1→2)]-glucopyranoside, 3*′*-hydroxy-4*″*-omethylisoscutellarein-7-O-[6-acetyl-allosyl-(1→2)]-glucopyranoside, 4*′*-O-methylisoscutellarein-7-O-[allosyl-(1→2)]-glucopyranoside, 3*′*-hydroxy-4*′*-O-methylisoscutellarein-7-O-(6*‴*-acetylhexosyl)-hexoside, isoscutellarein-7-O-[6*″*,6*‴*-di-acetyl-allosyl(1→2)]-glucopyranoside, 3*′*-hydroxy-4*′*-O-methylisoscutellarein-7-O-[6*″*,6*‴*-di-acetylallosyl(1→2)]-glucopyranoside	Herbarium SASSA 1099	[118]
*Tanacetum audibertii*	Super critical fluid extract	Aerial parts	Trans-linalyl oxide acetate, artemisia ketone, 1,8-cineole, artemisyl acetate	Herbarium CAG 737/A	[76]
	Hydrodistillated essential oil	Aerial parts	Artemisia ketone, trans-linalyl oxide acetate and 1,8-cineole, artemisyl acetate	Herbarium CAG 737/A	[76]
	Hydroalcoholic extract	Aerial parts	Valine, alanine, aspartic acid, sucrose, α-glucose. Β-glucose, trigonelline, formic acid. Phenolic and flavonoids contents	Herbarium CAG 737/A	[119]
*Thymus herba-barona* subsp. *herba-barona*	Diethyl ether extract *	Aerial parts	Flavanones (eriodictyol, naringenin) and flavones (luteolin, sorbifolin, thymusin, cirsiliol, apigenin, sideritoflavone, cirsimaritin, cirsilineol, xanthomicrol, 8-methoxycirsilineol and genkwanin)	N.A.	[243]
	Essential oil *	Aerial parts	Eight chemotypes (thymol, carvacrol, linalool, geraniol, α-terpenyl acetate, terpinen-4-ol, carvone and cis-dihydrocarvone)	N.A.	[67,190]
	Essential oil	Aerial parts	Differences between two sites (thymol, p-cymene, γ-terpinene, linalool; thymol, carvacrol, p-cymene, γ-terpinene borneol)	N.A.	[78]
	Essential oil	Aerial parts	Carvacrol, borneol, p-cymene	N.A.	[79]
	Essential oil	Aerial parts	Linalool, carvacrol	Herbarium CAG 1065	[244]
	Essential oil	Aerial parts	Carvacrol, thymol	Herbarium CAG 1065	[77]
*Verbascum conocarpum* subsp. *conocarpum*	Ethanolic extract	Aerial parts	One iridoid (aucubin) and a phenyl-propanoid compound (verbascoside)	Herbarium CAG 27-2	[237]
*Vinca difformis* subsp. *sardoa*	N.A.	Roots	Alkaloids (norfluorocurarine, akuammigine, carapanaubine, majdine, isomajdine, rauvoxinine, *ent*-N(1)-methyl-14,15-didehydroaspidospermidine **, N(1)-methyl-14,15-didehydroaspidofractinine **, N(1)-methylaspidofractinine **, N(1)-methyl-14,15-didehydrotuboxenine **)	N.A.	[245]
	Aqueous acetic acid extract	Aerial parts	Indole alkaloids (conoflorine, N(1)-methyl-14,15-didehydro-12-hydroxyaspidofractinine **, N(1)-methyl-14,15-didehydro-12-methoxyaspidofractinine **, N(1)-formyl-14,15-didehydroaspidofractinine **, N(1)-formyl-14,15-didehydro-12-hydroxyaspidofractinine **, venalstonine and N(1)-methyl-14,15-didehydroaspidofractinine)	N.A.	[246]
	Ethanol extract	Aerial parts	Iridoid glucosides (loganic acid and loganin)	N.A.	[247]
	Methanolic extract	Leaves	Quinic acid, chlorogenic acid, caffeoylquinic acid isomer 1 and robinin	Herbarium SASSA 820	[140]
	Histochemical preparation	Roots, stems, petioles, leaves and flowers	Indole alkaloids	Herbarium CAG 1301 [248]	[248,249]

N.A. = information not available; * Samples collected in countries other than Sardinia; ** Compounds isolated for the first time from Sardinian endemic species.

### 3.4. Criticism and Future Perspectives

All aforementioned information underlined that Sardinian endemic species could be the inspiration for many valuable therapeutic agents, since they are a rich source of biologically active compounds with unique structures that could contribute toward modern drug discovery. This chemical diversity is the result of a long evolution and adaptation to a wide range of habitats, that have modified the biosynthetic pathways to provide those organisms with a survival advantage in the ecosystems they inhabit [250]. However, the majority of endemic species, which could also represent an economic potential in the identification of new bioactive agents, has not yet been studied. One of the major obstacles arises from the limitations of plant material, especially regarding ecologically rare and endangered species. Indeed, even though small amounts of plant material are usually needed for a preliminar biological evaluation, larger quantities are required for the identification and characterization of bioactive compounds. Considering that a large number of medicinal and aromatic plants utilized all over the world are threatened [251] and two thirds of them are directly collected in the wild, it is necessary to promote and plan a rational exploitation of the plant biodiversity as a source of new bioactive substances. This observation is even more true for the endemic species, since they are particularly sensitive due to their restricted distribution and the small and often fragmented population size [15,16,17,252,253,254]. Thus, additional routes for resupply plant material need to be considered and, among the feasible strategies, the ex situ cultivation of threatened medicinal plants represents an encouraging protective approach in order to reconcile the conservation needs with research in the pharmaceutical field [19,255]. This approach may be pursued for endemic plants that require conservation efforts to prevent or mitigate the extinction risk. As recently demonstrated, the multiplication in nursery for several Mediterranean threatened endemic plants was an easy procedure, not cost-expensive and a practicable solution to obtain a large number of new individuals for several uses [16,256].

In addition to their ex situ cultivation, biotechnological strategies could also be pursued for the preservation and sustainable valorization of endemic plants, as well as their economic development [257]. Indeed, biotechnologies allow for producing bioactive molecules through in vitro cultivation or micropropagation of valuable species, as is well-documented by Giamperi and coauthors [222] for *Glechoma sardoa*.

## 4. Conclusions

This review provided, for the first time, an up-to-date and comprehensive overview on ethnobotanical uses, biological properties and phytoconstituents of Sardinian endemic plants, with the aim to supply input for future research prospects. Taking into account our results, only 17% of Sardinian endemic species have been subjected to phytochemical and/or pharmacological evaluation, with a great potential in the pharmaceutical field. Many of these exhibited significant antimicrobial, antinflammatory, antiviral, antioxidant and anticancer properties, even though these properties are not correlated to their traditional uses. Indeed, our literature survey pointed out that the selection of endemic plants for extract screening has probably been achieved by chemotaxonomic and biodiversity-driven approaches. Besides species intensively studied, such as *Helichrysum italicum* subsp. *thyrrenicum*, *Santolina insularis*, *Hypericum hircinum* subsp. *hircinum* and *Salvia desoleana*, there are many others deserving of attention and in-depth evaluation. They represent a great source of phytoconstituents, often unique and structurally diverse, due to adaptation to the local environment. It is worth noting that, in spite of their great value as sources of bioactive compounds, the majority of Sardinian endemic species have not been investigated, representing a potential to be explored in the identification of new bioactive agents. However, endemic species are often threatened and vulnerable, due to their rareness, and thus they need to be conserved and protected. One of the major critical issues in studying endemic species is represented by the limited availability of plant material necessary for biological evaluation and characterization of bioactive compounds. Therefore, additional strategies, such as ex situ or in vitro cultivation, need to be considered in order to reconcile the safeguard interest with research in the pharmaceutical field.

## Figures and Tables

**Table 1:** List of Sardinian endemic species for which ethnobotanical, phytochemical and/or pharmacological data have been published (1965–2020). For each taxon, the distribution range was reported by using the following acronyms: SA (Sardinia); CO (Corsica); AT (Tuscan Archipelago); BL (Balearic Islands); HI (Hyères Island); LI (Liguria); TO (Tuscany); SI (Sicily); TN (Tunisia).

Family	Taxon	Distribution Range
Amaryllidaceae	*Pancratium illyricum* L.	SA-CO-AT
Apiaceae	*Ferula arrigonii* Bocchieri	SA-CO
	*Seseli praecox* (Gamisans) Gamisans	SA-CO
Apocynaceae	*Vinca difformis* Pourr. subsp. *sardoa* Stearn	SA
Araceae	*Arum pictum* L.f. subsp. *pictum*	SA-CO
Aristolochiaceae	*Aristolochia tyrrhena* E.Nardi & Arrigoni	SA-CO-AT
Asteraceae	*Artemisia caerulescens* L. subsp. *densiflora* (Viv.) Kerguélen & Lambinon	SA-CO
	*Centaurea horrida* Badarò	SA
	*Helichrysum italicum* (Roth) G.Don subsp. *tyrrhenicum* (Bacch., Brullo & Giusso) Herrando, J.M.Blanco, L.Sáez Galbany	SA-CO
	*Helichrysum saxatile* Moris subsp. *saxatile*	SA
	*Plagius flosculosus* (L.) Alavi & Heywood	SA-CO-AT
	*Ptilostemon casabonae* (L.) Greuter	SA-CO-HI
	*Santolina corsica* Jord. & Fourr.	SA-CO
	*Santolina insularis* (Gennari *ex* Fiori) Arrigoni	SA
	*Tanacetum audibertii* (Req.) DC.	SA-CO
Boraginaceae	*Borago morisiana* Bigazzi & Ricceri	SA
	*Borago pygmaea* (DC.) Chater & Greuter	SA-CO-AT
Euphorbiaceae	*Euphorbia hyberna* L. subsp. *insularis* (Boiss.) Briq.	SA-CO-LI-TO
	*Euphorbia pithyusa* L. subsp. *cupanii* (Guss. *ex* Bertol.) Radcl.-Sm.	SA-CO-SI
	*Euphorbia semiperfoliata* Viv.	SA-CO
Fabaceae	*Astragalus verrucosus* Moris	SA
	*Bituminaria morisiana* (Pignatti & Metlesics) Greuter	SA-TN
	*Genista arbusensis* Vals.	SA
	*Genista bocchierii* Bacch., Brullo & Feoli Chiapella	SA
	*Genista cadasonensis* Vals.	SA
	*Genista corsica* (Loisel.) DC.	SA-CO
	*Genista ephedroides* DC.	SA-CO
	*Genista morisii* Colla	SA
	*Genista pichisermolliana* Vals.	SA
	*Genista sulcitana* Vals.	SA
Hypericaceae	*Hypericum hircinum* L. subsp. *hircinum*	SA-CO-AT
	*Hypericum scruglii* Bacch., Brullo & Salmeri	SA
Lamiaceae	*Glechoma sardoa* (Bég.) Bég.	SA
	*Mentha requienii* Benth. subsp. *requienii*	SA-CO
	*Salvia desoleana* Atzei & V.Picci	SA
	*Stachys corsica* Pers.	SA-CO
	*Stachys glutinosa* L.	SA-CO-AT
	*Thymus herba-barona* Loisel. subsp. *herba-barona*	SA-CO
Orobanchaceae	*Euphrasia nana* (Rouy) Prain	SA-CO
Plantaginaceae	*Cymbalaria muelleri* (Moris) A.Chev.	SA
	*Linaria flava* (Poir.) Desf. subsp. *sardoa* (Sommier) A.Terracc.	SA-CO
Plumbaginaceae	*Limonium contortirameum* (Mabille) Erben	SA-CO
	*Limonium morisianum* Arrigoni	SA
Polygalaceae	*Polygala sardoa* Chodat	SA
Ranunculaceae	*Staphisagria requienii* (DC.) Spach subsp. *picta* (Willd.) Peruzzi	SA-CO-BL-HI
Rubiaceae	*Galium corsicum* Spreng.	SA-CO
	*Galium glaucophyllum* Em.Schmid	SA
	*Galium schmidii* Arrigoni	SA
Scrophulariaceae	*Scrophularia trifoliata* L.	SA-CO-AT
	*Verbascum conocarpum* Moris subsp. *conocarpum*	SA-CO-AT
Urticaceae	*Urtica atrovirens* Req. *ex* Loisel.	SA-CO-AT

## Supplementary Materials

The following are available online at https://www.mdpi.com/2223-7747/9/8/958/s1: Table S1: Biological activities of Sardinian endemic species.
