# Supplementary material for: So Uncommon and so Singular, but Underexplored: An Updated Overview on Ethnobotanical Uses, Biological Properties and Phytoconstituents of Sardinian Endemic Plants
Source: Plants (Basel). 2020 Jul 29;9(8):958. doi: 10.3390/plants9080958 (PMC7465485; doi:10.3390/plants9080958)
Supplement: Supplementary file 1 [file plants-09-00958-s001.pdf]

Table S1. Biological activities of Sardinian endemic species.

Taxon	Biological activities	Extract or bioactive compounds	Experimental method	References
Artemisia caerulescens subsp. *densiflora*	Antimicrobial	EO of aerial parts	Agar disc diffusion test (*Rhodotorula* spp., *Candida* spp, *Aspergillus* spp., *Alternaria* spp., *Fusarium* spp.)	[46]
	Antioxidant	EO of aerial parts	DPPH, inhibition of lipid oxidation and hypochlorous acid tests	[98]
	Cytotoxic	EO of aerial parts	MTT assay (A375, MDA-MB231 and HCT116 human tumor cell lines)	[98]
Astragalus verrucosus	Antimicrobial	astraverrucins II**	Agar disc diffusion test (*Staphylococcus aureus*, *Enterobacter agglomerans*, *Escherichia coli*, *Salmonella infantis*, *Pseudomonas aeruginosa*, *Aspergillus niger*, *Botrytis cinerea*)	[48]
Bituminaria morisiana	Antioxidant	Erybraedin C, plicatin B	Autoxidation and iron or EDTA-mediated oxidation of linoleic acid, Cholesterol oxidation assays, TBARS method, oxidative damage induced in VERO cells by FeCl3	[130]
	Cytotoxic	Pet and EthAc extracts of leaves	MTT assay (VERO cells)	[102]
Euphorbia pithyusa subsp. *cupanii*	Antioxidant	Seeds fatty acids	DPPH and β-carotene bleaching tests	[131]
Euphorbia semiperfoliata	Antiviral	EthAc extract of whole plant/ jatropane diterpenes** and 4-deoxyphorbol esters**	Virus-cell-based assay in VERO cells (Chikungunya virus strain 899, Sindbis virus strain HRsp, and Semliki Forest virus strain Vietnam); MTT assay in MT-4 cells (HIV-1 strain (IIIB) and HIV-2 strain (ROD)	[81,82]
	Antimicrobial	Jatrophane esters	Transport assays by monitoring Nile Red accumulation in cells overexpressing Cdr1p (AD-CDR1) or Mdr1p (AD-MDR1) of *Candida albicans*	[50]
	Anticancer	Three jatrophane polyesters	*In vitro* polymerization assay and electron microscopy; cell viability (Balb/c 3T3 fibroblasts, MCF-7 breast cancer cells and Caco-2 colon carcinoma cells); morphological analysis using the trypan blue exclusion assay and an inverted phase-contrast microscope, fluorescence microscopy, flow cytometry, Western blotting	[104]
	Antioxidant	Seeds fatty acids	DPPH and β-carotene bleaching tests	[131]
Ferula arrigonii	Anticancer	2α-Hydroxiferutidin, ferutidin, lapiferin, Jaeskeanadiol	Growth-inhibitory experiments (COLO 320HSR, LS-174T, and WiDr human colon cancer cell lines); Clonogenic assay; flow cytometry; Type-ll EBS analysis in cell lines	[105]
Genista cadasonensis	Antioxidant	EtOH extract of aerial parts	DPPH, ABTS radical cation decolourisation assays	[132]
Genista ephedroides	Antigenotoxic	Licoflavone C	Micronucleus (MN) assay on stimulated and cytochalasin B-blocked human lymphocytes	[125]
	Estrogenic	Licoflavone C	Yeast reporter gene assay (*Saccharomyces cerevisiae* RMY326 ER-ERE); yeast based estrogen receptor assay	[164,165]
Genista morisii	Estrogenic	Daidzein, genistein, isoprunetin	Yeast reporter gene assay (*Saccharomyces cerevisiae* RMY326 ER-ERE)	[164]
	Anti estrogenic	Luteolin	Yeast based estrogen receptor assay	[165]
Glechoma sardoa	Antimicrobial	EO of aerial parts	Agar dilution technique (*Staphylococcus aureus* ATCC 25923, *Staphylococcus epidermidis* of clinical isolation, *Escherichia coli* ATCC 25922, *Pseudomonas aeruginosa* ATCC 27853)	[51]
Helichrysum italicum subsp. *tyrrhenicum*	Antimicrobial	EO of aerial parts	Paper test (*Botrytis cinerea, Cercospora beticola, Fusarium oxysporum lycopersici, Fusarium graminearum, Helminthosporium oryzae, Pythium ultimum, Pyricularia orizae, Rhizoctonia solani, Sclerotium rolfsii, Phytophthora capsici* and *Septoria tritici*); fungicide activity (MIC); broth microdilution test (*Escherichia coli* ATCC 8739, *Staphylococcus aureus* ATCC 6538, *Pseudomonas aeruginosa* ATCC 9027, *Candida albicans* ATCC 10231, *Aspergillus brasiliensis (niger)* ATCC 16404, *Candida* spp. clinical strains)	[52,53]

		MeOH extract of aerial parts; OM; arzanol	Broth microdilution test (*Staphylococcus aureus* subsp. *aureus* ATCC 29213, *Enterococcus faecalis* ATCC, 29212, *Micrococcus luteus* ATCC9622, *Proteus mirabilis* ATCC 29852, *Proteus vulgaris* ATCC 6361, *Escherichia coli* ATCC 4350, *Pythium ultimum*, *Trichophyton mentagrophytes* var. *mentagrophytes*)	[54,57,59]
	Cytotoxic	EO; arzanol	MTT assay (MDA-MB 231, HCT-116, A375, Caco-2, HeLa human tumor cell lines and B16F10 mouse melanoma cells); AlamarBlue test (differentiated Caco-2 cells)	[97,101]
	Antioxidant	MeOH extract of aerial parts; arzanol and helipyrone	DPPH, ABTS, β-carotene bleaching tests; cholesterol oxidation assay; liposomes oxidation assay; Fe-NTA model of *in vivo* oxidative stress; determination of 5-LO product formation in cell-free systems and in intact cells; LDL oxidation assay; cell lipid peroxidation assay	[54,97,101,133,135]
	Antinflammatory	Act extract of aerial parts; arzanol	Luciferase assay (Jurkat cells); isolation of human peripheral monocytes and determination of IL-6, TNF-α, IL-1-β, and PGE2; determination of 5-LO product formation in cell-free systems and in intact cells; determination of COX-1 activity in platelet homogenates and COX-2 activity in monocyte homogenates; determination of COX-1 product formation in washed platelets; determination of 6-keto PGF1α formation in intact A549 cells; SDS–PAGE and Western blot; determination of PGE2, 6-keto PGF1α, and TXB2 formation in human whole blood; preparation of crude mPGES-1 in microsomes of A549 cells and determination of PGE2 synthase activity; carrageenan-induced pleurisy in rats	[83,133]
	Antiviral	Act extract of aerial parts; arzanol	Production of VSV-pseudotyped recombinant viruses and infection assays	[83]
Hypericum hircinum subsp. *hircinum*	MAO inhibitor	quercetin	Forced Swimming Test Procedure (FST)	[182]
	Antiviral	betulinic acid, 5,7,3′,5′-tetrahydroxyflavanone-7-O-glucoside	RNase H polymerase-independent cleavage assay; DNA polymerase assay; Evaluation of $MgCl_2$ chelation	[84]
	Antioxidant	EtOH extract of aerial parts	DPPH, ABTS, FRAP-Ferrozine (FZ) assays	[136,137]
	Anti-collagenase	EtOH extract of aerial parts	Spectrophotometric collagenase assay	[136]
	α-glucosidase inhibitor	HydOH and EtOH extracts of aerial parts	Spectrophotometric α-glucosidase assay	[137]
	Cardioprotective	EtOH extract of aerial parts	Recording changes in ECG, heart rate and measuring the levels of cardiac marker enzymes – lactic acid dehydrogenase, creatine phosphokinase, glutamic oxaloacetic transaminase, the antioxidant defence enzymes – reduced glutathione (GSH), superoxide dismutase (SOD) and lipid peroxidative value (LPO) in animal model	[178]
	Tyrosinase inhibitor	MW extract of aerial parts	Spectrophotometric tyrosinase inhibitory assay	[159]
	Elastase inhibitor	MW extract of aerial parts	Spectrophotometric elastase inhibitory assay	[159]
Hypericum scruglii	α-glucosidase inhibitor	HydOH and EtOH extracts of aerial parts	Spectrophotometric α-glucosidase assay	[137]
	Antivira	3-geranyl-1-(2'-methylbutanoyl)phloroglucinol, 3-geranyl-1-(2'-methylpropanoyl)phloroglucinol, 3-(13-hydroxygeranyl)-1-(2'-methylbutanoyl)phloroglucinol**, quercitrin	Expression and purification of recombinant HIV-1 RT, IN and LEDGF; RNase H polymerase-independent cleavage assay; RDDP assay; Homogeneous time resolved fluorescence (HTRF) LEDGF dependent assay; phenotypic analyses with fully replicating recombinant HIV-1 strain; time of addition test	[86]
	Elastase inhibitor	MW extract of aerial parts	Spectrophotometric elastase inhibitory assay	[159]
	Antioxidant	HydOH and EtOH extracts of aerial parts	DPPH, ABTS, FRAP-ferrozine, β-carotene bleaching and deoxyribose assays	[137]
	Fibromyalgia	EtOH extract incapsulated in liposomes	*In vivo* e *in vitro* experiments (details not available)	[181]

Limonium contortirameum	α-glucosidase inhibitor	H_20 extract of aerial parts	*In vitro* enzymatic starch digestion assay	[174]
	α-amylase inhibitor	H_20 extract of aerial parts	*In vitro* enzymatic starch digestion assay	[174]
	Pancreatic lipase inhibitor	H_20 extract of aerial parts	Spectrophotometric quantification of PL activity and assaying PL inhibition	[174]
Limonium morisianum	Antiviral	MeOH extract of aerial parts	Expression and purification of recombinant HIV-1 RT, IN and LEDGF; RNase H polymerase-independent cleavage assay; Homogeneous time resolved fluorescence (HTRF) LEDGF dependent assay	[88]
	Antiviral	Myricetin, (-)-epigallocatechin 3-O-gallate	Fluorescence-based EBOV r-VP35-dsRNA interaction assay; molecular docking	[90]
	Tyrosinase inhibitor	MW extract of aerial parts	Spectrophotometric tyrosinase inhibitory assay	[159]
	Elastase inhibitor	MW extract of aerial parts	Spectrophotometric elastase inhibitory assay	[159]
	Antimicrobial	MW extract of aerial parts	Broth microdilution test (*Staphylococcus aureus* ATCC 25293, *S. epidermidis* ATCC 12,228), *Escherichia coli* (ATCC 25,922) and *Klebsiella pneumoniae* (ATCC 9591); cell viability assay on African green monkey kidney cells (Vero ATCC CCL-81)	[60]
Mentha requienii subsp. *requienii*	Antimicrobial	EO of aerial parts	Agar disc diffusion test (*Rhodotorula* spp., *Candida albicans*, *Aspergillus fumigatus*, *Fusarium* spp., *Alternaria* spp. and a mixture of moulds)	[47]
Pancratium illyricum	Anticancer	Ungeremine	Monitoring the relaxing activity of human topoisomerase I (hTopoI) and IIα (hTopoIIα) enzymes on supercoiled pBR322; alkaloid-DNA complex formation	[61]
	Antimicrobial	Ungeremine	Monitoring the relaxing activity of human topoisomerase I (hTopoI) and IIα (hTopoIIα) enzymes on supercoiled pBR322; alkaloid-DNA complex formation	[61]
		MeOH extract of bulbs; lycorine	Broth microdilution method (30 clinical *Candida albicans* strains)	[62]
	Acetylcholinesterase inhibitor	11α-hydroxy-O-methylleucotamine**	Microplate Acetylcholinesterase (AChE) assay	[185]
	Anti-collagenase	MeOH extract of bulbs	Spectrophotometric collagenase assay	[62]
Plagius flosculosus	Antinflammatory	EthAc extract of aerial parts	Inhibition of induced IL-6 expression; NF-*k*B-dependent reporter gene activity screen; LPS-induced expression of IL-1, IL-6, TNF, and PGE2 in primary human monocytes (ELISA); inhibitory effects on TNFa-induced luciferase activity in Jurkat 5.1 cells	[149]
	Cytotoxic	DCM extract of leaves	WST-1-based cell viability assay on CD4+ Jurkat human leukemia T-cells (ATCC TIB-152 and Bcl-2 transfected cells) and HL-60 cells (ATCC CCL-240 cells); determination of apoptosis by FACS (fluorescence-activated cell sorting) experiments with HL-60 cells	[109]
	Antimicrobial	SFC extract and EO of leaves	Kirby-Bauer method of agar diffusion (*Staphylococcus aureus* ATCC 25923, *Escherichia coli* ATCC 25922, *Enterococcus faecalis* CA 3908, *Bacillus subtilis*, *Candida albicans* CDC B385 and *Mycobacterium smegmatis* CIP 103599); broth microdilution in casitone broth method (*Botrytis cinerea* 644 and *Rhizoctonia solanii*)	[64]
Salvia desoleana	Antimicrobial	EO of leaves	Macrodilution and microdiution methods (*Aspergillus niger* ATCC 6275, *A. ochraceus* ATCC 12066, *A. versicolor* ATCC 11730, *A. flavus* ATCC 9170, *A. terreus* ATCC 16792, *Alternaria alternata* ATCC 13963, *Penicillium ochrochloron* ATCC 9112, *P. funiculosum* ATCC 10509, *Cladosporium cladosporioides* ATCC 13276, *Trichoderma viride* IAM 5061, *Fusarium tricinctum* CBS 514478, *Phomopsis helianthin* ATCC 201540, *Trichophyton entagrophytes*, *T. rubrum*, *T. tonsurans*, *Epidermophyton floccosum*, *Microsporum cannis*)	[65]
		EO of leaves	Dilution technique (MIC) on *Staphylococcus a*ureus ATCC 25923, *Staphylococcus epidermidis* of clinical isolation, *Streptococcus salivarius* ATCC 13419, *Streptococcus sanguis* ATCC 10556), *Escherichia coli* ATCC25922, *Pseudomonas aeruginosa* ATCC27853, *Klebsiella pneumoniae*, *Citrobacter freundii*, *Enterobacter cloacae*, *Serratia marcescens* of clinical isolation, *Candida albicans* ATCC10231	[66]

	Antioxidant	SFC extract of leaves	Measurement of intracellular reactive oxygen species (ROS molecular probe H_2DCFDA) on human endothelial cells (HUVECs and ECV304); measurement of cell proliferation (analysis of the cellular DNA content using CyQUANT® NF assay kit)	[138]
	Anti-inflammatory	EO of leaves	Carrageenan induced hind paw oedema test in male albino Wistar rats	[150]
	Analgesic	EO of leaves	Antinociceptive test on Swiss mice (inhibition of writhings induced by formic acid injection)	[150]
	Permeation through buccal mucosa	Linalyl acetate, α-terpinil acetate, 1,8-cineole, α-terpineol, linalool, β-pinene	In vitro diffusion experiments in standard Franz diffusion cells	[184]
	Antiviral	EO of leaves	Plaque reduction assay on Vero cells (inhibition of HSV-1, HSV-2 and RSV); time of addition test; viral plaque assay (virus inactivation assay)	[91]
Santolina corsica	Antinflammatory	EO of leaves	Structural analysis of samples of bronchoalveolar lavage (BAL) with optical microscope (MO); ultra-structural analysis of BAL with an electron microscope (TEM)	[151]
		N-hex extract of aerial parts	Inhibition of NO production in LPS-stimulated RAW 264.7 murine macrophages (Griess reagent)	[110]
	Anticancer	MeOH extract of aerial parts	Cell viability assay by MTT assay (A549, HeLa, PC3, MCF7, MDA-MB-231 and MCF 10 A cell lines)	[110]
	Antioxidant	MeOH extract of aerial parts and *N*-hex of aerial parts	DPPH, ABTS, β-carotene bleaching, FRAP tests	[110]
	Pro-apoptotic	MeOH extract of aerial parts	Wound-healing scratch assay; migration assay; invasion assay; TUNEL assay; morphological analysis (MDA-MB-231 cells)	[110]
	Antimicrobial	EO of aerial parts	Agar diffusion method (*Staphylococcus aureus* ATCC 6538P, *Escherichia coli* ATCC 11775, *Pseudomonas aeruginosa* CIP A22, *Enterobacter aerogenes* ATCC 13048, *Campylobacter jejuni* F38O11, *Listeria innocua* ATCC 33090); Broth dilution method to evaluate MIC and MBC; time-kill studies; bacteriolysis test	[67–69]
Santolina insularis	Antiviral	EO of aerial parts and liposome-incorporated EO	Plaque reduction assay on Vero cells (HSV-1 and HSV-2); yield reduction assay; inhibition of plaque development assay; attachment, penetration, and post attachment virus neutralization assays	[92,93]
	Antimicrobial	EO of aerial parts	Agar dilution method (*Staphylococcus aureus* ATCC25923, *Escherichia coli* ATCC25922, *Candida albicans* 5M, *Candida tropicalis* CA44, *Cryptococcus neoformans* CA1.<br>Broth macrodilution methods (MIC and MLC) on *Candida albicans* ATCC 10231, *C. tropicalis* ATCC 13803, *C. krusei* H9, *C. guillermondii* MAT23, *C. parapsilosis* ATCC 90018, *Cryptococcus neoformans* CECT 1078, *Trichophyton mentagrophytes* FF7, *T. rubrum* CECT 2794, *T. mentagrophytes* var. *interdigitale* CECT 2958, *T. verrucosum* CECT 2992, *Microsporum canis* FF1, *M. gypseum* CECT 2908, *Epidermophyton floccosum* FF9, *Aspergillus niger* ATCC 16404, *A. fumigatus* ATCC 46645, *A. flavus* F44; effect on *C. albicans* germ tube formation.<br>Broth microdilution technique on *Propionibacterium acnes* ATCC 6919 (in presence of sub-inhibitory concentration of chitosan)	[70,71,73]
		EO-loaded vesicles	Vesicle characterization by cryogenic transmission electron microscopy (cryo-TEM); stability of vesicles study by turbiscan optical analyser (TurbiScanLab®); vesicles structure study by Small/Wide-Angle X-ray Scattering (SAXS and WAXS); *ex vivo* skin penetration/permeation studies to the skin using pig skin by Franz diffusion cells	[74]
	Cytotoxic	Two germacrane sesquiterpenes	Measurement of mitochondrial respiration (mitochondrial-dependent reduction of MTT to formazan) on Caco-2 (human colon carcinoma) and peritoneal macrophages cells	[116]
	Anti-inflammatory	MeOH extract of leaves and EO of aerial parts	*In vivo* croton oil-induced dermatitis assay (Male CD-1 mice)	[152]

		EO of aerial parts	NO production assay in mouse leukemic macrophage cell line (RAW 264.7) using the Griess reagent; expression of the pro-inflammatory proteins iNOS and COX-2	[71]
Stachys glutinosa	Antimicrobial	EO of aerial parts	Kirby Bauer method (*Aeromonas hydrophyla* and *A. sobria*, *Candida albicans*, *C. glabrata*, *C. kruseii*, *C. parapsilosis*, *Enterococcus faecalis* (ATCC 24212), *Escherichia coli* (ATCC 35218), *Klebsiella pneumonie* (ATCC 700603), *Rodotorula rubra*, *Staphylococcus aureus* ATCC 43300, *S. epidermidis*, *Streptococcus* group D), *Vibrio alginolyticus* and *V. cholerae*	[75]
	Opioid receptor antagonist	Xanthomicrol	Receptor binding assays [^{3}H]-DAMGO−[^{3}H]-DPDPE (Opioid Receptors); Tail-Flick test model of antinociception on male CD1 mice	[186]
	Antioxidant	EtOH extract of aerial parts	ABTS, DPPH, β-carotene/linoleic acid assays; scavenging of hydrogen peroxide, superoxide anion-scavenging (SAS) ($\cdot O_2^-$) and HOCl scavenging assays	[118]
	Antiproliferative	EtOH extract of aerial parts	MTT assay (HepG2, MCF7 and C2C12 cell lines)	[118]
Tanacetum audibertii	Antimicrobial	EO of aerial parts	Macrodilution broth method (*C. krusei* H9, *C. guillermondii* MAT23, *C. albicans* ATCC 10231, *C. tropicalis* ATCC 13803, *C. parapsilopsis* ATCC 90018, *Cryptococcus neoformans* CECT 1078, *Epidermophyton floccosum* FF9, *Trichophyton mentagrophytes* FF7, *Microsporum canis* FF1, *T. mentagrophytes* var. *interdigitale* CECT 2958, *T. rubrum* CECT 2794, *T. verrucosum* CECT 2992, and *M. gypseum* CECT 2908)	[76]
	Anticancer	HydOH extract of aerial parts	MTT assay on Human osteosarcoma cells U2OS and Human umbilical vein endothelial cells (HUVEC); cell cycle analysis by cytofluorimetric analysis; analysis of p53 levels by flow cytometry; caspase activity assay by colorimetric CaspACE assay system	[119]
Thymus herba-barona subsp. *herba-barona*	Antimicrobial	EO of aerial parts	Macrodilution broth method (*Candida krusei* H9, *C. guillermondii* MAT23, *C. albicans* ATCC 10231, *C. tropicalis* ATCC 13803, *C. parapsilopsis* ATCC 90018, *Cryptococcus neoformans* CECT 1078, *Aspergillus flavus* F44, *A. niger* ATCC 16404, *A. fumigatus* ATCC 46645, *Epidermophyton floccosum* FF9, *Microsporum canis* FF1, *M. gypseum* CECT 2908, *Trichophyton mentagrophytes* FF7, *T. mentagrophytes var. interdigitale* CECT 2958, *T. rubrum* CECT 2794, *T. verrucosum* CECT 2992).<br>Agar diffusion method (*Staphylococcus aureus* ATCC 6538P, *Escherichia coli* ATCC 11775, *Pseudomonas aeruginosa* CIP A22, *Enterobacter aerogenes* ATCC 13048 and *Campylobacter jejuni* F38O11).<br>Determination of MIC and MBC by broth microdilution method (*E. coli* ATCC 25922, *E. coli* O157:H7 ATCC 35150, *Ps. aeruginosa* ATCC 27853, *S. aureus* ATCC 25923, *S. epidermidis* ATCC 12228, *E. faecalis* ATCC 29212, *Yersinia enterocolitica* ATCC 9610, *Candida albicans* ATCC 10231, *B. cereus* ATCC 11778, *Listeria monocytogenes* ATCC 7644, *Salm. typhimurium* ATCC 14028 and *Sacc. cerevisiae* ATCC 9763).<br>Determination of MIC by agar dilution technique (*Bacillus* spp., *Staphylococcus aureus* ATCC 25923, ATCC 6538, *S. epidermidis* of clinical isolates, *Streptococcus salivarius* ATCC 13419, *S. sanguis* ATCC 10556, *Escherichia coli* ATCC 25922, *Pseudomonas aeruginosa* ATCC 27853, *Klebsiella pneumoniae*, *Citrobacter freundii*, *Enterobacter cloacae* clinical isolates)	[67,77-79]
	Cytotoxic	EO of aerial parts	MTT assay on mouse macrophage cell line, Raw 264.7 (ATCC number: TIB-71)	[77]
Vinca difformis subsp. *sardoa*	Antioxidant	MeOH extract of leaves	DPPH and ABTS assays	[140]

In the column "Extract or bioactive compound" the type of extract has been abbreviated as follows: Act (acetone); EO (essential oil); DCM (dichloromethane); EtOH (ethanol); EthAc (ethyl acetate); HydOH (hydro alcoholic); H_2O (aqueous); MetOH (methanol); MW (methanol-water); N-hex (n-hexane); OM (oil macerate); Pet (petroleum ether); SFC (super critical fluid extract).

**Compound identified for the first time from Sardinian endemic species
